# Neonatal Diet Impacts the Large Intestine Luminal Metabolome at Weaning and Post-Weaning in Piglets Fed Formula or Human Milk

**DOI:** 10.3389/fimmu.2020.607609

**Published:** 2020-12-07

**Authors:** Fernanda Rosa, Katelin S. Matazel, Anne K. Bowlin, Keith D. Williams, Ahmed A. Elolimy, Sean H. Adams, Lars Bode, Laxmi Yeruva

**Affiliations:** ^1^ Arkansas Children’s Nutrition Center, Little Rock, AR, United States; ^2^ Department of Pediatrics, University of Arkansas for Medical Sciences, Little Rock, AR, United States; ^3^ Arkansas Children’s Research Institute, Little Rock, AR, United States; ^4^ Department of Microbiology and Immunology, University of Arkansas for Medical Sciences, Little Rock, AR, United States; ^5^ Department of Biostatistics, University of Arkansas for Medical Sciences, Little Rock AR, United States; ^6^ Larsson-Rosenquist Foundation Mother-Milk-Infant Center of Research Excellence, University of California San Diego, La Jolla, CA, United States; ^7^ Department of Pediatrics, University of California San Diego, La Jolla, CA, United States

**Keywords:** human milk, infant formula, neonates, metabolism, host-microbiota

## Abstract

The impact of human milk (HM) or dairy milk-based formula (MF) on the large intestine’s metabolome was not investigated. Two-day old male piglets were randomly assigned to HM or MF diet (n = 26/group), from postnatal day (PND) 2 through 21 and weaned to a solid diet until PND 51. Piglets were euthanized at PND 21 and PND 51, luminal contents of the cecum, proximal (PC) and distal colons (DC), and rectum were collected and subjected to metabolomics analysis. Data analyses were performed using Metaboanalyst. In comparison to MF, the HM diet resulted in higher levels of fatty acids in the lumen of the cecum, PC, DC, and rectum at PND 21. Glutamic acid was greater in the lumen of cecum, PC, and DC relative to the MF group at PND 21. Also, spermidine was higher in the DC and rectal contents of HM relative to MF at PND 21. MF diet resulted in greater abundances of amino acids in the cecal lumen relative to HM diet at PND 21. Additionally, several sugar metabolites were higher in various regions of the distal gut of MF fed piglets relative to HM group at PND 21. In contrast, at PND 51, in various regions there were higher levels of erythritol, maltotriose, isomaltose in HM versus MF fed piglets. This suggests a post weaning shift in sugar metabolism that is impacted by neonatal diet. The data also suggest that infant diet type and host-microbiota interactions likely influence the lower gut metabolome.

## Introduction

Human milk (HM) contains a diversity of bioactive components including lipids, human milk oligosaccharides (HMOs), a variety of cytokines, and microbiota that can influence the child’s development, immune function, and microbiota colonization during early life (1–3). Although studies have indicated the positive impact of HM diet on immune function (4, 5), microbiota composition (6), and child’s growth (7), mechanisms behind these outcomes are poorly understood due to limitations associated with gut sample collection from infants. During early life, cow’s milk-based formula (MF) has been chosen as an alternative to human milk (8), but the degree to which MF feeding alters the gastrointestinal tract (GI) milieu relative to HM remains to be fully characterized.

The use of omics technologies such as metagenomics and metabolomics provide platforms to gain new insights about the mechanisms underlying diet-associated differences in the infant’s growth and overall health during the neonatal period. For instance, microbiota analysis of infant’s stool demonstrated that HM diet shapes microbiota colonization and enriches bacterial species *Bifidobacteria* and *Bacteroides* during exclusive HM feeding relative to formula diet (9, 10). Furthermore, previous studies using metabolomics investigated fecal and serum metabolite profiles of HM versus MF fed infants (11–14). While providing valuable insights, the GI bioregional aspects of HM and MF feeding have remained difficult to study.

We and others reported the use of animal models (primate and piglets) to investigate the impact of MF diet on gut microbiota, immune system, and metabolism (15–22). These models are valuable tools to explore the effects of neonatal regimes on gastrointestinal tract development and maturation (18, 23–25), since they allow the collection of multiple tissues and GI regions for large scale analysis which is limited in human studies (26). Our group developed a piglet model under controlled conditions (i.e., an isocaloric diet of HM or MF, vivarium housing), and have demonstrated that HM-fed piglets had a higher abundance of *Bacteroides* which is similar to the microbiota composition of breast-fed infants (17). Most recently, using the same piglet model our group reported that formula diet could alter the epithelial barrier integrity through disruption of tight junctions in the small intestine of formula-fed piglets compared to the HM-fed (18). These findings are indicative that a piglet model is a promising tool to evaluate the influence of neonatal diet on gut metabolism. Here, we present a comparative metabolomics analysis of the distal gastrointestinal tract of piglets fed HM or MF diet during the first 21 days of life and post-weaning neonatal diet at day 51.

## Materials and Methods

### Experimental Design

The animal study was conducted in accordance with the ethical guidelines for animal research approved by the Institutional Animal Care and Use Committee at the University of Arkansas for Medical Sciences. The detailed experimental design as well as the diet composition were previously published (19). Briefly, White Dutch Landrace Duroc male piglets within 2-d old were randomly assigned to two groups (n = 26/group), fed an isocaloric diet of HM (Mother’s Milk Bank of North Texas), or a dairy-based MF (milk formula; Similac Advance powder; Ross products, Abbott Laboratories, Columbus, OH) to meet the nutrient requirements of growing pigs as per the guidelines published by the National Research Council (NRC) (27). At postnatal day (PND) 14 complementary food (i.e., solid pellets) (starter pellets; Teklad, TD 140608; Harlan Laboratories) was introduced to the piglets and weaned to *ad libitum* solid pellets from PND21 to PND51 (19). Piglets were immunized on PND 21 and PND 35 with oral administration of 100 µg of cholera toxin (C8052, Millipore Sigma) and 100 µg of cholera toxin subunit B (CTB; C9903, Millipore Sigma). Piglets also received The DAPTACEL [diphtheria, tetanus, pertussis (DTaP)] vaccine (0.5 mL; Arkansas Children’s Hospital pharmacy) by intramuscular injection. Control piglets received vehicle.

### Tissue Collection

At PND 21 and 51 piglets were euthanized after anesthetization with isoflurane, followed by exsanguination. Cecum, proximal colon, distal colon, and rectum contents were collected within a scintillation vial by pinching the tissue and sliding the constriction toward the open end. All samples were immediately snap-frozen in liquid nitrogen and stored at −80°C until further analysis.

### Metabolite Profiling and Statistical Analyses

Cecum, PC, DC, and rectum contents were subjected to metabolomics analyses using gas chromatography/mass spectrometry (GC/MS) at the West Coast Metabolomics Center at University of California Davis. Approximately 4 mg of contents from experimental samples from each region were used to have a pool for quality control (QC) during the process of the metabolome data. Detailed GC/MS instrument conditions were reported previously (28). Briefly, a total of 0.5 µL of each sample was injected splitless into an Agilent 6890 GC equipped with a Gerstel automatic liner exchange system (ALEX) that includes a multipurpose sample (MPS2) dual rail, and a Gerstel CIS cold injection system (Gerstel, Muehlheim, Germany). The gas chromatograph was controlled using Leco ChromaTOF software. Constituted of helium mobile phase, the gas flow rate through a 30 m long, 0.25 mm i.d. Rtx-5Sil MS column (0.25 μm 95% dimethyl 5% diphenyl polysiloxane film) with additional 10 m integrated guard column (Restek, Bellefonte PA) was 1 mL/min. The transfer line temperature between gas chromatograph and mass spectrometer was set to 280°C. Electron impact was generated by a 70-eV ionization and with an ion source temperature of 250°C. Acquisition rate is 17 spectra/second, with a scan mass range of 85–500 Da. Compounds were identified by comparison with Fiehn lab BinBase database annotations (29), database identifier [i.e., InChI key (30)], the compound annotation metadata (i.e., retention index, quantification mass, BinBase identifier, and mass spectrum), and PubChem annotation (31). A list of peak heights, retention time and mass to charge (m/z) were obtained. 549 metabolites were detected in all samples, including 282 annotated and 267 unknown (non-annotated) metabolites. The unknown metabolites were excluded from the current analysis. The raw data was processed and analyzed in MetaboAnalyst 4.0 (32). On postnatal day 51, diet and immunization interactions were assessed by Permutational multivariate ANOVA (PERMANOVA) with 999 permutations (**Supplemental Table 1**). No Diet × immunization interaction was observed for cecum (P > 0.25), PC and DC (P ≥ 0.42), and for rectum content metabolites (P = 0.11). Therefore, control and immunized data were pooled in the analysis of the PND 51. The QC samples were subjected to multivariate analysis in MetaboAnalyst to check the precision of the metabolomics analysis. The supervised partial least squares discriminant analysis (PLS-DA) score plot for the QC samples (**Supplemental Figure 1**) showed the tight clustering of the QC samples indicating the precise outcome from the metabolites process. Metabolites peak intensities were normalized by the sum of all identified metabolites (33) and log transformed prior to multivariate statistical analysis (34). The PLS-DA score plots were used to see the overall difference between metabolite profiles of HM and MF groups followed by Pattern Hunter analysis in MetaboAnalyst to detect the significant differences in metabolites between groups. A metabolite was considered to be statistically different when *P* - value ≤ 0.05, Benjamini-Hochberg adjusted false discovery rate (FDR) ≤ 0.15, and variable importance in projection (VIP) score > 1.0 (34, 35). Based on the identification of the significantly altered metabolites in HM and MF-fed groups, we calculated the fold change (FC) for each metabolite.

## Results

### MF Diet-Fed Piglets Have a Distinct Metabolite Profile in the Distal Gastrointestinal Tract Relative to HM Fed Piglets at PND 21

**Figure 1:**
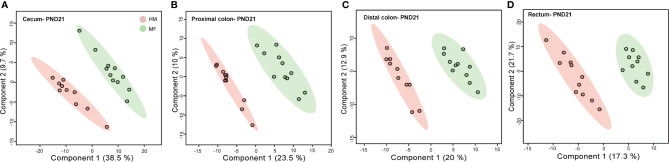
Two-dimensional scores plot of partial least squares discriminant analysis (PLS-DA) model showing how distal gut content abundances of annotated metabolites can discriminate human milk (HM) versus milk formula (MF) feeding groups during the neonatal period in piglets. Panels depict **(A)** cecum **(B)**, proximal colon **(C)**, distal colon, and **(D)** rectal contents at postnatal day (PND) 21. PLS-DA scores (i.e., individual piglet scores) for PLS-DA components (dimensions) 1 and 2 are displayed. Shadows with color are 95% confidence regions. Pink circles indicate individual HM-fed piglets and green circles indicate MF-fed piglets. Sample numbers were n = 8–11 per group.

Previously we have demonstrated that microbiota changes were predominant in the large intestine of piglets fed the MF diet relative to the HM group (17). Thus, to evaluate the impact of early diet on the large intestine metabolome, the cecum, proximal colon, distal colon, and rectum contents were examined at PND 21. The PLS-DA model of metabolite showed robust separation of dietary groups at PND 21 in cecal, PC, DC, and rectal regions of the gastrointestinal tract (**Figures 1A–D**).

### Metabolite Profile in Different Regions of the Distal Gastrointestinal Tract at PND 21 Is Impacted by Neonatal Diet

At PND 21, within the lumen of large intestine and rectum, a total of 123 cecal, 111 PC, 95 DC, and 62 rectal metabolites from diverse chemical classes including fatty acids, amino acids, lipids, carbohydrates, vitamins, steroids, and co-metabolites were significantly different between HM and MF diet-fed piglets (**Tables 1** **–** **7** and **Supplemental Table 2**). The complete list of all detected metabolites (including non-annotated “unknown” metabolites) within each intestinal region is presented in the **Supplementary Table 6**.

**Table 1:** Average abundances [quantifier ion (quantion) intensities] of fatty acids significantly different when comparing human milk (HM) or milk formula (MF) diet groups, in cecum, proximal colon, distal colon, and rectum contents of piglets at postnatal day (PND) 21.

Cecum	HM^1^	SEM^2^	MF^1^	SEM^2^	FC^3^	P^4^	FDR^5^	VIP^6^
Myristic acid	145,457	21,036	73,124	18,704	1.99	0.01	0.03	1.2
Palmitic acid	625,873	39,506	444,733	43,594	1.41	0.01	0.02	1.23
Linolenic acid	16,553	2,620	6,639	1,319	2.49	<0.01	0.02	1.24
Linoleic acid	9,148	1,503	3,055	604	2.99	<0.01	0.01	1.43
Oleic acid	58,553	22,709	7,959	1,415	7.36	<0.01	0.01	1.31
Palmitoleic acid	1,581	214	604	89	2.62	<0.01	<0.01	1.6
Stearic acid	4,829,607	212,505	3,878,986	321,597	1.25	0.03	0.06	1.03
**Proximal colon**								
Myristic acid	332,535	78,155	121,609	23,150	2.73	<0.01	0.01	1.55
Palmitic acid	1,127,510	94,825	752,618	45,928	1.5	<0.01	0.01	1.55
Linolenic acid	32,957	3,956	17,855	4,297	1.85	0.02	0.06	1.2
Linoleic acid	32,011	5,977	11,235	2,916	2.85	<0.01	0.01	1.51
Oleic acid	159,855	62,469	39,707	21,273	4.03	0.02	0.06	1.18
Palmitoleic acid	4,320	1,317	785	112	5.51	<0.01	<0.01	1.91
Cis-gondoic acid	3,097	327	2,050	225	1.51	0.03	0.09	1.08
**Distal colon**								
Myristic acid	700,211	64,821	291,343	48,434	2.4	<0.01	0	1.87
Palmitic acid	2,023,370	165,035	1,354,469	78,078	1.49	<0.01	0	1.73
Linolenic acid	75,731	14,902	18,827	4,014	4.02	<0.01	0.01	1.68
Oleic acid	469,449	50,482	73,856	23,936	6.36	<0.01	0	2
Palmitoleic acid	8,349	1,340	615	72	13.57	<0.01	<0.01	2.6
Cis-gondoic acid	5,677	977	2,122	345	2.68	<0.01	0.01	1.65
Stearic acid	8,584,424	666,445	10,187,891	291,167	0.84	0.05	0.15	1.06
**Rectum**								
Myristic acid	632,851	53,966	401,123	85,521	1.58	0.01	0.07	1.51
Palmitic acid	1,515,125	64,253	1,102,436	96,584	1.37	<0.01	0.02	1.79
Linolenic acid	67,665	12,297	21,882	3,716	3.09	0.01	0.06	1.57
Linoleic acid	54,138	8,348	13,835	3,649	3.91	<0.01	<0.01	2.05
Oleic acid	440,191	80,906	85,398	47,031	5.15	<0.01	0.01	1.89
Palmitoleic acid	8,349	1,340	615	72	13.57	<0.01	<0.01	2.6

^1^Mean of normalized (mTIC) peak intensities (mz/rt) for human milk (HM) or milk formula (MF) after MetaboAnalyst analyses; n=8–11/group.

^2^SEM, Standard error of the mean.

^3^Fold change of HM mean to MF mean.

^4^P-value ≤ 0.05.

^5^FDR, Benjamini-Hochberg adjusted P-value.

^6^VIP, Variable importance in projection in PLS-DA models using all annotated metabolites to compare HM and MF within each bio-region. The table only presents metabolites with significant differences between diet groups; all detected metabolites are provided in **Supplementary Table 6**.

**Table 2:** Average abundances [quantifier ion (quantion) intensities] of polyamines significantly different when comparing human milk (HM) or milk formula (MF) diet groups, in cecum, proximal colon, distal colon, and rectum contents of piglets at postnatal day (PND) 21.

Cecum	HM^1^	SEM^2^	MF^1^	SEM^2^	FC^3^	P^4^	FDR^5^	VIP^6^
Putrescine	4,460	3,457	5,720	1,288	0.78	0.03	0.07	1.01
**Distal Colon**								
Spermidine	58,259	7,924	14,837	7,484	3.93	<0.01	0.01	1.62
**Rectum**								
Spermidine	23,474	6,506	4,243	3,592	5.53	<0.01	0.04	1.65

^1^Mean of normalized (mTIC) peak intensities (mz/rt) for human milk (HM) or milk formula (MF) after MetaboAnalyst analyses; n=8–11/group.

^2^SEM, Standard error of the mean.

^3^Fold change of HM mean to MF mean.

^4^P-value ≤ 0.05.

^5^FDR, Benjamini-Hochberg adjusted P-value.

^6^VIP, Variable importance in projection in PLS-DA models using all annotated metabolites to compare HM and MF within each bio-region. The table only presents metabolites with significant differences between diet groups; all detected metabolites are provided in **Supplementary Table 6**.

**Table 3:** Average abundances [quantifier ion (quantion) intensities] of sugar metabolites significantly different when comparing human milk (HM) or milk formula (MF) diet groups, in cecum, proximal colon, distal colon, and rectum contents of piglets at postnatal day (PND) 21.

Cecum	HM^1^	SEM^2^	MF^1^	SEM^2^	FC^3^	P^4^	FDR^5^	VIP^6^
Galactose-6-phosphate	82	11	216	39	0.38	<0.01	<0.01	1.46
Glucose-1-phosphate	1,059	240	2,373	262	0.45	<0.01	<0.01	1.48
Raffinose	157	34	328	95	0.48	0.03	0.07	1.01
Glycerol	231,576	20,963	340,232	34,945	0.68	0.02	0.05	1.08
Isomaltose	428	59	717	60	0.60	<0.01	0.01	1.37
Maltotriose	356	81	1,456	515	0.24	0.02	0.05	1.08
Ribitol	1,465	195	2,561	325	0.57	0.02	0.05	1.07
**Proximal colon**								
Galactitol	5,648	2,174	1,427	613	3.96	<0.01	0.02	1.46
Galactose-6-phosphate	153	21	373	80	0.41	0.01	0.03	1.34
Glycerol	400,598	34,375	568,545	43,853	0.7	<0.01	0.02	1.4
Raffinose	180	28	303	42	0.6	0.02	0.08	1.13
**Distal colon**								
1,5-anhydroglucitol	2,825	495	1,337	156	2.11	<0.01	0.02	1.54
Galactitol	8,608	3,342	882	76	9.76	<0.01	<0.01	1.96
Sorbitol	12,441	4,608	3,973	518	3.13	0.01	0.06	1.29
Fructose	8,678	1,031	5,426	1,139	1.6	0.03	0.1	1.19
Xylulose	7,403	984	3,784	569	1.96	<0.01	0.02	1.49
Ribose	271,496	42,458	143,274	20,425	1.89	0.01	0.03	1.43
Galactose-6-phosphate	136	22	354	80	0.38	<0.01	0.02	1.53
Raffinose	157	17	248	34	0.63	0.01	0.06	1.31
**Rectum**								
1,5-anhydroglucitol	2,209	130	1,674	224	1.32	0.02	0.12	1.36
Maltotriose	247	33	391	53	0.63	0.02	0.1	1.4
Mannose	5,318	867	9,690	1,390	0.55	0.02	0.1	1.4

^1^Mean of normalized (mTIC) peak intensities (mz/rt) for human milk (HM) or milk formula (MF) after MetaboAnalyst analyses; n=8–11/group.

^2^SEM, Standard error of the mean.

^3^Fold change of HM mean to MF mean.

^4^P-value ≤ 0.05.

^5^FDR, Benjamini-Hochberg adjusted P-value.

^6^VIP, Variable importance in projection in PLS-DA models using all annotated metabolites to compare HM and MF within each bio-region. The table only presents metabolites with significant differences between diet groups; all detected metabolites are provided in **Supplementary Table 6**.

**Table 4:** Average abundances [quantifier ion (quantion) intensities] of amino acids significantly different when comparing human milk (HM) or milk formula (MF) diet groups, in cecum, proximal colon, distal colon, and rectum contents of piglets at postnatal day (PND) 21.

Cecum	HM^1^	SEM^2^	MF^1^	SEM^2^	FC^3^	P^4^	FDR^5^	VIP^6^
Histidine	2,041	607	4,831	639	0.42	<0.01	0.01	1.43
Valine	47,321	11,157	121,492	16,114	0.39	<0.01	0.01	1.42
Leucine	68,267	14,347	118,450	16,248	0.58	0.01	0.04	1.12
Isoleucine	39,144	7,645	81,579	12,819	0.48	0.01	0.02	1.22
Methionine	6,886	1,252	11,264	1,274	0.61	0.01	0.04	1.12
Taurine	75	5	152	23	0.49	<0.01	0.01	1.31
Cysteine	832	135	2,285	382	0.36	<0.01	0.01	1.4
Glutamic acid	611,642	67,690	383,277	44,281	1.6	0.03	0.07	1
**Proximal colon**								
Cysteine	3,074	561	7,987	1,215	0.38	<0.01	<0.01	1.7
N-acetylornithine	1,295	171	2,047	236	0.63	0.03	0.09	1.1
Glutamic acid	1,176,854	153,757	697,884	65,464	1.69	0.01	0.05	1.25
N-acetylaspartic acid	24,555	7,547	12,064	4,117	2.04	0.02	0.07	1.16
**Distal colon**								
Cysteine	1,494	229	2,757	403	0.54	0.01	0.06	1.3
Glutamic acid	930,473	150,262	306,803	36,781	3.03	<0.01	0	1.86
N-acetylaspartic acid	24,116	10,159	5,426	869	4.44	0.02	0.07	1.27
**Rectum**								
Histidine	6,240	1,424	14,434	3,220	0.43	0.03	0.14	1.32
Valine	236,629	26,908	517,077	87,043	0.46	<0.01	0.03	1.74
Leucine	262,738	27,431	588,107	113,109	0.45	0.01	0.05	1.61
Threonine	30,098	4,222	70,540	14,278	0.43	<0.01	0.04	1.64
Isoleucine	145,147	17,537	354,847	68,488	0.41	<0.01	0.04	1.66
Glycine	44,615	3,944	89,099	11,985	0.5	<0.01	0.02	1.85
Proline	71,923	9,809	235,145	56,370	0.31	<0.01	0.03	1.75
Methionine	21,104	3,049	53,916	13,521	0.39	0.01	0.08	1.49
Phenylalanine	48,286	6,454	108,093	25,076	0.45	0.03	0.13	1.33
N-acetylornithine	1,798	312	974	238	1.85	0.02	0.11	1.38
Glutamic acid	521,372	106,688	246,722	34,239	2.11	0.01	0.08	1.46
N-acetylaspartic acid	10,420	3,025	3,625	1,075	2.87	0.01	0.06	1.55

^1^Mean of normalized (mTIC) peak intensities (mz/rt) for human milk (HM) or milk formula (MF) after MetaboAnalyst analyses; n=8–11/group.

^2^SEM, Standard error of the mean.

^3^Fold change of HM mean to MF mean.

^4^P-value ≤ 0.05.

^5^FDR, Benjamini-Hochberg adjusted P-value.

^6^VIP, Variable importance in projection in PLS-DA models using all annotated metabolites to compare HM and MF within each bio-region. The table only presents metabolites with significant differences between diet groups; all detected metabolites are provided in **Supplementary Table 6**.

**Table 5:** Average abundances [quantifier ion (quantion) intensities] of cholesterol and bile acids significantly different when comparing human milk (HM) or milk formula (MF) diet groups, in cecum, proximal colon, distal colon, and rectum contents of piglets at postnatal day (PND) 21.

Cecum	HM^1^	SEM^2^	MF^1^	SEM^2^	FC^3^	P^4^	FDR^5^	VIP^6^
Cholesterol	8,019	1,200	30,126	3,223	0.27	<0.01	<0.01	1.79
Deoxycholic acid	1,040	193	7,030	1,706	0.15	<0.01	<0.01	1.62
**Proximal Colon**								
Cholesterol	6,901	883	23,671	2,835	0.29	<0.01	<0.01	1.88
Deoxycholic acid	1,570	393	4,101	852	0.38	0.02	0.06	1.2
Chenodeoxycholic acid	37,595	13,813	89,407	29,531	0.42	0.02	0.08	1.13
**Distal Colon**								
Cholesterol	18,311	3,627	49,675	4,448	0.37	<0.01	0	1.9
Deoxycholic acid	2,647	713	11,300	1,845	0.23	<0.01	0.01	1.63
Chenodeoxycholic acid	33,830	11,018	82,652	30,280	0.41	0.04	0.13	1.12
**Rectum**								
Deoxycholic acid	2,805	974	7,852	1,377	0.36	<0.01	0.04	1.68

^1^Mean of normalized (mTIC) peak intensities (mz/rt) for human milk (HM) or milk formula (MF) after MetaboAnalyst analyses; n=8–11/group.

^2^SEM, Standard error of the mean.

^3^Fold change of HM mean to MF mean.

^4^P-value ≤ 0.05.

^5^FDR, Benjamini-Hochberg adjusted P-value.

^6^VIP, Variable importance in projection in PLS-DA models using all annotated metabolites to compare HM and MF within each bio-region. The table only presents metabolites with significant differences between diet groups; all detected metabolites are provided in **Supplementary Table 6**.

**Table 6:** Average abundances [quantifier ion (quantion) intensities] of tryptophan metabolites significantly different when comparing human milk (HM) or milk formula (MF) diet groups, in cecum, proximal colon, distal colon, and rectum contents of piglets at postnatal day (PND) 21.

Cecum	HM^1^	SEM^2^	MF^1^	SEM^2^	FC^3^	P^4^	FDR^5^	VIP^6^
Indole-3-propionic acid	2,155	539	5,569	989	0.39	<0.01	0.01	1.31
3-hydroxyphenylacetic acid	620	67	1,421	159	0.44	<0.01	<0.01	1.65
**Proximal Colon**								
3-hydroxyphenylacetic acid	884	137	1,806	306	0.49	0.01	0.05	1.24
**Distal Colon**								
Tryptophan	24,762	4,056	13,072	3,373	1.89	0.01	0.05	1.35
5-hydroxy-3-indoleacetic acid	776	80	344	77	2.25	<0.01	0.01	1.69
**Rectum**								
5-hydroxy-3-indoleacetic acid	824	87	429	83	1.92	<0.01	0.02	1.79

^1^Mean of normalized (mTIC) peak intensities (mz/rt) for human milk (HM) or milk formula (MF) after MetaboAnalyst analyses; n=8–11/group.

^2^SEM, Standard error of the mean.

^3^Fold change of HM mean to MF mean.

^4^P-value ≤ 0.05.

^5^FDR, Benjamini-Hochberg adjusted P-value.

^6^VIP, Variable importance in projection in PLS-DA models using all annotated metabolites to compare HM and MF within each bio-region. The table only presents metabolites with significant differences between diet groups; all detected metabolites are provided in **Supplementary Table 6**.

**Table 7:** Average abundances [quantifier ion (quantion) intensities] of sugar metabolites (erythritol, lyxose, xylitol, xylose, pentose, xylulose, ribose, maltotriose, isomaltose), tryptophan metabolites (indole-3-propionic acid), and fatty acids (behenic acid) significantly different when comparing human milk (HM) or milk formula (MF) diet groups, in cecum, proximal colon, distal colon, and rectum contents of piglets at postnatal day (PND) 51.

Cecum	HM^1^	SEM^2^	MF^1^	SEM^2^	FC^3^	P^4^	FDR^5^	VIP^6^
Erythritol	1,445	356	761	104	1.9	0.03	0.78	2.02
Indole-3-propionic acid	12,716	2,080	7,397	1,240	1.72	0.03	0.78	2.07
**Distal Colon**								
Erythritol	1,116	255	652	40	1.71	0.05	0.39	1.57
Lyxose	19,364	3,196	9,660	950	2	<0.01	0.16	2.41
Xylitol	2,899	245	1,950	118	1.49	<0.01	0.16	2.36
Xylose	282,684	50,219	135,380	17,049	2.09	<0.01	0.16	2.25
Pentose	74,638	22,946	27,458	3,058	2.72	<0.01	0.16	2.25
Xylulose	12,922	1,177	8,456	1,028	1.53	0.01	0.22	2.03
Ribose	364,271	36,115	250,569	34,238	1.45	0.03	0.39	1.73
Behenic acid	65,712	3,150	54,919	3,179	1.2	0.02	0.3	1.87
**Rectum**								
Erythritol	655	32	432	41	1.52	<0.01	0.08	2.96
Maltotriose	586	117	201	14	2.91	<0.01	0.3	2.53
Isomaltose	706	116	445	40	1.59	0.03	0.65	1.9
Behenic acid	40,727	1,532	35,181	1,820	1.16	0.03	0.65	1.92

^1^Mean of normalized (mTIC) peak intensities (mz/rt) for human milk (HM) or milk formula (MF) after MetaboAnalyst analyses; n=9–15/group.

^2^SEM, Standard error of the mean.

^3^Fold change of HM mean to MF mean.

^4^P-value ≤ 0.05.

^5^FDR, Benjamini-Hochberg adjusted P-value.

^6^VIP, Variable importance in projection in PLS-DA models using all annotated metabolites to compare HM and MF within each bio-region. The table only presents metabolites with significant differences between diet groups; all detected metabolites are provided in **Supplementary Table 6**.

### Fatty Acids and Polyamines Had Higher Abundances in the Distal Gut of HM Relative to MF Fed Piglets at PND21

The fatty acids myristic, palmitic, linolenic, linoleic, oleic, and palmitoleic were the common metabolites identified throughout the lumen of cecum, PC, DC, and rectum at PND 21, which had greater abundance in the HM than in the MF group. In the lumen of cecum, the saturated fatty acid stearic acid was greater in the HM-fed group relative to the MF group (**Table 1**). In the PC and DC of HM fed piglets, the fatty acids cis-gondoic acid was higher relative to the MF group (**Table 1**). In addition, the fatty acids cis-gondoic had greater abundance in the DC lumen of HM than MF-fed piglets (**Table 1**). Spermidine was another metabolite common to the DC and rectal lumen that was higher in the HM compared to the MF-fed piglets (**Table 2**). However, Putrescine was lower in HM cecal lumen in comparison to MF group.

### Carbohydrates and Amino Acids Were Higher in MF Fed Piglets Relative to HM Group at PND 21

The carbohydrates 1, 5-anhydroglucitol, galactitol, sorbitol, and fructose were greater in the DC contents of HM-fed relative to MF-fed piglets, while the carbohydrates galactose-6-phosphate and raffinose had greater abundances in the cecal, PC, and DC lumen of MF relative to HM-fed piglets (**Table 3**). Isomaltose, ribitol, and maltotriose were greater in the cecal contents of MF relative to the HM group. In addition, 1, 5-anhydroglucitol, mannose and maltotriose were higher in rectal contents in MF group relative to HM group. The essential amino acids histidine, valine, and leucine were greater in the cecal lumen and rectal contents of MF-fed piglets relative to the HM group (**Table 4**). Additionally, threonine, isoleucine, and phenylalanine were greater in the rectal contents of the MF-fed group compared to HM-group. While the non-essential amino acids glycine and proline were greater in the rectal contents, and taurine and cysteine were greater in the cecal contents of MF-fed compared to the HM-fed piglets. In rectal contents, a higher abundance of the amino acids N-acetylornithine, and N-acetylaspartic acid was observed in the HM group (**Table 3**). However, glutamic acid was higher in the HM lumen of cecal, PC, and DC while N-acetyl aspartic acid was higher in PC, DC and rectal contents relative to MF-fed piglets.

### Cholesterol and Bile Acids Were Higher in MF Diet-Fed Piglets at PND 21

Cholesterol was significantly higher in the MF group in cecal, PC, and DC lumen (**Table 5**). Interestingly, secondary bile acid deoxycholic acid had greater abundance throughout the 4 regions of the distal gut in comparison to HM-fed piglets. Also, the primary bile acid chenodeoxycholic acid was higher in the luminal contents of PC and DC in the MF group relative to the HM group.

### Tryptophan Metabolites Were Impacted by Neonatal Diet in the Large Intestine at PND 21

The metabolites indole-3-propionic acid and 3-hydroxyphenylacetic acid had greater abundance in MF-fed piglets relative to the HM group in the cecal lumen. Within the DC lumen, 5-hydroxy-3-indoleacetic acid and tryptophan were higher in the HM than in the MF group. Additionally, the tryptophan metabolite 5-hydroxy-3-indoleacetic acid was greater in the rectum of the HM relative to the MF group (**Table 6**).

### At PND 51 the Metabolite Profile in the Distal Gastrointestinal Tract Is Less Distinct and Showed a Lower Number of Metabolite Differences Between HM and MF

**Figure 2:**
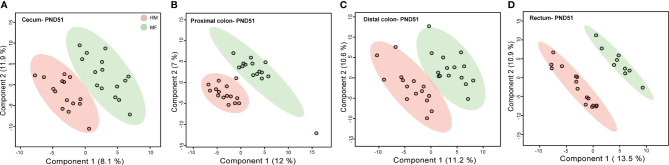
Two-dimensional scores plot of partial least squares discriminant analysis (PLS-DA) model showing how distal gut content abundances of annotated metabolites can discriminate human milk (HM) versus milk formula (MF) feeding groups during the neonatal period in piglets. Panels depict **(A)** cecum **(B)**, proximal colon **(C)**, distal colon, and **(D)** rectal contents at postnatal day (PND) 51. PLS-DA scores (i.e., individual piglet scores) for PLS-DA components (dimensions) 1 and 2 are displayed. Shadows with color are 95% confidence regions. Pink circles indicate individual HM-fed piglets and green circles indicate MF-fed piglets. Sample numbers were n = 9–15 per group.

PLS-DA plots demonstrated that the distribution of metabolites had less separation between HM and MF groups at PND 51 (**Figures 2A–D**), except for the rectal contents that had a robust separation of the metabolite profile between HM and MF groups. At PND 51 between HM and MF fed piglets, 15 metabolites were significantly different in cecum and PC, 37 in DC, and 21 in the rectum by using the *P* < 0.05 and a VIP > 1.0 criteria (**Supplemental Table 3**). The lumen of the cecum of HM fed piglets had higher abundance of indole-3-propionic acid relative to the MF-fed piglets. The sugar alcohol erythritol was a common metabolite in the cecum, DC, and rectum, with higher abundance in the HM group in comparison to the MF group. Additionally, behenic acid was a common fatty acid in the DC and rectal lumen which was higher in the HM-fed relative to the MF-fed piglets at PND 51 (**Table 7**).

### Serum Metabolome Impacted by Neonatal Diet at PND 21 and 51

At PND 21, serum metabolome revealed higher abundance of threonic acid and cysteine in the MF relative to the HM fed group. While palmitoleic acid was higher in the HM group. At PND 51, the HM diet resulted in greater abundances of sugar metabolites including maltotriose and xylitol, and greater indole-3-propionic acid relative to MF-fed group. The complete list of serum metabolites impacted by HM and MF diets are presented in the **Supplemental Table 4**.

## Discussion

The present study provides metabolite profiles in the cecum, colon, and rectal lumen of HM versus MF feeding regimens in a porcine model at PND 21 and PND 51. We found that diet has a pronounced effect on metabolite profiles in the lumen of the cecum, PC, DC, and rectum at PND 21 (pre-weaning) but an attenuated effect at PND 51 (~1-month post-weaning). We observed a greater number of metabolite changes in the luminal region of the cecum of HM-fed piglets compared to the MF group at PND 21. A greater abundance of fatty acids and polyamines was observed in HM, while amino acids were higher in MF at PND 21. The persistent effect of the neonatal diet was observed at PND 51 with altered sugar metabolism in HM versus MF fed piglets.

Of particular note was the observation that HM feeding impacted tryptophan metabolism differently than MF feeding, at PND 21. The majority of ingested protein is digested and absorbed by the small intestine (36); however, a significant amount of proteins and amino acids may reach the colon, which is degraded by different microbial species (37). Amino acids in the lower gut may also derive in part from the host (e.g., sloughed tissue, mucous, and epithelial cells from the lining of the intestines) (38–40). In the lumen of DC, tryptophan was higher in the HM-fed group. In addition, a derivative of indole-3-acetic acid (IAA), 5-hydroxy-3-indole acetic acid, was greater in the DC and rectum of HM-fed piglets. Interestingly, we have shown that IAA concentration was also higher in the feces of HM-fed infants at 3 months of age in comparison to formula fed infants (41). *Bacteroides* genera have been shown to convert tryptophan to indole-3-acetic acid. In support of this notion, we have reported a higher abundance of genera *Bacteroides* in infants fed human milk and a higher abundance of genera from class Bacteroidia in the rectal lumen of HM fed piglets (17, 41). These results suggest that tryptophan in the HM group is likely metabolized by distal gut microbiota. In addition, bioactive microbial tryptophan metabolites, indole, indole-3-propionic acid, and IAA have been reported to modulate inflammatory response by promoting IL-22 production in the gastrointestinal tract of mice through the activation of aryl hydrocarbon receptor (AhR) (42, 43). We speculate that the higher tryptophan metabolite levels with human milk feeding promotes the interaction with the host-microbiota which might dampen inflammation.

Neonatal diet also resulted in a divergent fatty acid profile at PND 21 in the large intestine. The human milk lipid profile is variable, and several factors including maternal age, lactation stage, metabolic disorders, maternal diet, among others can modulate the lipid composition (44). HM is composed of more than 200 fatty acids including high levels of oleic and linoleic acids, and these are likely obtained from the mother’s diet (45). Essential fatty acids such as linoleic and linolenic cannot be synthesized by the mammalian body from the precursor oleic acid due to the lack of specific enzymes (Δ12 and Δ15-desaturase and hydrogenase), thus adequate intake of these fatty acids through dietary regimens are needed (46). Furthermore, the fatty acid composition of monogastric animals (i.e., piglets) also depends on the dietary intake of fatty acids (47). In our study, throughout the 4 regions evaluated (from cecum to rectum) the linolenic and linoleic essential fatty acids were higher in the HM fed piglets relative to MF at PND 21. Additionally, other fatty acids, myristic, palmitic, oleic, and palmitoleic were common metabolites identified throughout the large intestine of HM-fed relative to the MF-fed group. Studies from our laboratory and others identified higher circulating fatty acids in the HM group. For example, palmitoleic acid was higher in HM-fed serum in comparison to MF-fed piglets (**Supplemental Table 4**), and free fatty acids such as palmitic acid, oleic acid, and stearic acid were higher in the plasma of infants fed HM relative to formula-fed (11). It is suggestive that fatty acids are delivered to infants from HM and in part from the mother’s diet. Dietary fatty acids have been shown to exert immunomodulatory effects during inflammatory conditions in humans (48) and in mouse models (49, 50). For example, linolenic acid had an anti-inflammatory effect by decreasing the secretion of the pro-inflammatory IL-6 in an intestinal model using the Caco-2-cell line (51). Additionally, essential fatty acids have been shown to be transferred from sow milk into the piglets’ enteric tissues, which might play a role in the immune response and in the epithelial integrity (52). For instance, polyunsaturated fatty acids supplementation to pregnant sows resulted in lower markers of inflammation in the post weaning period of piglets (53). These data, suggest that fatty acids from mothers’ milk exhibit immune protection to infants.

Human milk contains low levels of putrescine compared to spermine and spermidine in term and preterm milk (54). Interestingly, we observed a significantly lower level of putrescine in the lumen of the cecum while spermidine was significantly higher in the lumen of DC and rectum in HM relative to MF. It is possible that HM is the source for these polyamines observed in the distal gut and may provide benefits to infants by various mechanisms. For example, spermine and spermidine play a role in the maintenance of the colonic (55) and intestinal mucosa in mammals (56). Spermidine is considered essential for postnatal intestinal maturation and it has been reported to be higher in human milk than in formulas (57, 58). In addition, spermidine supplementation suppresses inflammatory DC function and systemic inflammation in the psoriasis mouse model (59). Interestingly, human infants fed dairy-based formula had greater levels of the pro-inflammatory molecules (IL8 and IL1β) in the feces compared to HM-fed infants at 1-month (60) and our most recent report suggested higher inflammatory status in MF than HM fed piglets (18). In addition, spermidine has been shown to play a role in autophagy to rejuvenate memory B cell response in older individuals (61). Reduced B cell function causes poor vaccination efficacy and likely a higher incidence of infections. Several studies have demonstrated that HM fed infants have stronger vaccine response and lower respiratory tract infections during the infancy period (1, 2, 62–64). Moreover, in the same piglets we observed stronger vaccine response in HM versus MF fed piglets (19). Also, infant formula supplemented with polyamines increased the number of *Bifidobacterium* species in the large intestine of mice resulting in greater mucin production (65). Thus, the greater level of spermidine upon human milk feeding may benefit the infants by maintaining colon health, microbiota composition, and immune function.

While human milk cholesterol content varies from 90 to 150 mg/L, infant formulas have lower cholesterol content between 20–40 mg/L originated from dairy milk fat (66). Adequate cholesterol dietary intake is essential, especially for growing infants, for the production of steroid hormones, brain development, and lipoprotein metabolism (67, 68). However, a balance between cholesterol absorption and synthesis is required for maintaining whole-body cholesterol homeostasis (69). Formula-fed infants (70, 71) and piglets (24, 72, 73) have been shown to have higher hepatic cholesterol synthesis and fecal bile acid excretion. Fecal sterol excretion followed by intestinal breakdown can be associated with reduced intestinal absorption of cholesterol (68). In the current piglet study, the greater cholesterol detected in the cecum and colon contents of the MF group might be associated with a feedback mechanism (e.g., increased cholesterol synthesis) in response to the low dietary cholesterol uptake. In addition, the cholesterol synthesized in the liver is converted to primary bile acids such as cholic acid (CA), and chenodeoxycholic acid (CDCA) (74). These primary bile acids synthesized from cholesterol in hepatocytes are conjugated to the amino acids taurine or glycine for further biliary secretion (75). In our study, the greater abundance of the bile acids CDCA in the PC and DC lumen was associated with higher levels of amino acids taurine and glycine in the cecal contents of the MF group. In the distal colon, solely gut bacterial bile salt hydrolase (BSH) deconjugates bile acids to form the secondary bile acids deoxycholic acid (DCA) and lithocholic acid (LCA) (76). Importantly, we observed higher DCA in all 4 regions of the distal gut with MF diet suggesting as one of the mechanisms of maintaining cholesterol homeostasis is likely by excretion of secondary bile acids. The implications of a high level of cholesterol and bile acids in the gut can be speculated based on previously published literature (77). For example, bile acids can regulate the epithelial barrier integrity through activation of the farnesoid X receptor (FXR) on intestinal epithelial cells (74). DCA has been shown to induce gut dysbiosis, disrupt bile acid enterohepatic circulation, and promote intestinal inflammation (78). In addition, taurine has been shown to activate *Nlrp6* inflammasome and induce the release of the proinflammatory IL-18 by the intestinal epithelial cells (79). Moreover, the accumulation of DCA in the large intestine has been associated with passive absorption through the colon mucosa (76). Overall, these data suggest that cholesterol and bile acid homeostasis is impacted by the formula diet.

Glutamic acid (glutamate), glutamine, and taurine are the most abundant free amino acids (FAA) in human milk, accounting for approximately 50% of total FAA (80–82) while in dairy-based formulas taurine is the most prevalent FAA (83). In this study, throughout the distal gut regions, higher glutamic acid was detected in HM-fed piglets, likely derived from HM (82, 84). Glutamate intake through the HM diet might benefit the overall neonatal gut health since it has been reported to function as a major energy substrate for intestinal cells (84, 85). Thus, non-essential amino acids intake through human milk might supply infants with readily available nitrogen-compounds. Previous studies demonstrated that standard infant formulas have a lower concentration of free amino acid compared to breastmilk (80, 83) while hydrolysate formulas have a higher amount of amino acids relative to regular formulas (86). In our study, several amino acids (i.e., valine, cysteine, isoleucine, leucine, methionine, cysteine, glycine, histidine, and phenylalanine) were higher in the cecal and rectal contents of MF-fed piglets relative to HM at PND 21, likely due to higher amount of protein in formula. Interestingly, previous studies demonstrated higher levels of circulatory amino acids in formula-fed relative to breastfed infants likely due to higher protein intake with formula diet (11, 12, 87, 88). While we only observed higher cysteine levels in the serum of MF fed piglets (**Supplemental Table 4**), it is possible that in our piglets fasting conditions (8 h) were impacting the circulatory amino acid pool as most of the infant studies measured metabolites after 2–3 h of fasting (11).

Sugar metabolism was impacted by the formula diet relative to the HM diet in piglets. Several metabolites (UDP-glucuronic acid, lyxose, ribonic acid, maltrotriose, UDP-N-acetyl glucosamine, pyruvic acid, threonic acid, raffinose, melibiose, erythrose, xylulose, panose, maltose, mannose) were significantly higher in the MF group relative to the HM group in different regions of distal gut at 8 h of fasting. Interestingly, serum threonic acid (**Supplemental Table 4**) and urinary threonic acid, ribonic acid, and maltotriose (**Supplemental Table 5**) were also significantly higher in MF relative to HM piglets. Notably, galactose concentration was higher in infant formulas compared to mature human milk (89). In our piglet model MF diet has impacted the carbohydrate metabolism as observed by a higher abundance of galatcose-6-phosphate in the cecum and colon followed by higher glucose-1-phosphate in the cecum of MF-fed piglets at PND 21. Based on previous infant literature and our current data, it is suggestive that formula-fed piglets exhibited a trend to use more of the energy from carbohydrate while HM-fed piglets may use fat as the energy fuel during exclusive neonatal feeding (i.e., PND 21) (11, 13). Additionally, others demonstrated that carbohydrate intake was lower in breastfed infants at 3 and 6 months compared to formula-fed infants (90). Also, metabolites shared between urine and large intestine suggest that these could serve as biomarkers of host health and likely microbial metabolism.

Previous metabolomics studies of infants have shown that the introduction of complementary food minimizes metabolic profile differences in serum while there are clear metabolic changes upon exclusively HM or MF feeding in infants (11). Similarly, we observed less separation of metabolite profile at PND 51 between HM and MF fed piglets. However, sugar metabolites such as erythritol, lyxose, xylitol, xylose, pentose, xylulose, ribose, maltotriose, isomaltose were higher in HM fed relative to MF fed post-weaned piglets. In addition, maltotriose, xylitol followed a similar pattern in the serum of HM fed piglets (**Supplemental Table 4**) suggesting a shift toward carbohydrate metabolism in HM group post-weaning neonatal diet. Persistent effects on microbial metabolism of tryptophan to indole-3-propionic acid was also observed by a higher abundance of this metabolite in cecal lumen and serum of HM fed piglets (**Supplemental Table 4**).

## Limitations

The human milk fed to piglets was a pool from donors at 2 to 12 months of lactation, which is prone to variations on the milk composition including fatty acids. The different stages of lactation and the variability from the donor mothers might alter the distal tract metabolite profile. The components added to the HM and MF to maintain the requirement of a growing piglet may impact the luminal metabolome.

## Conclusions

**Figure 3:**
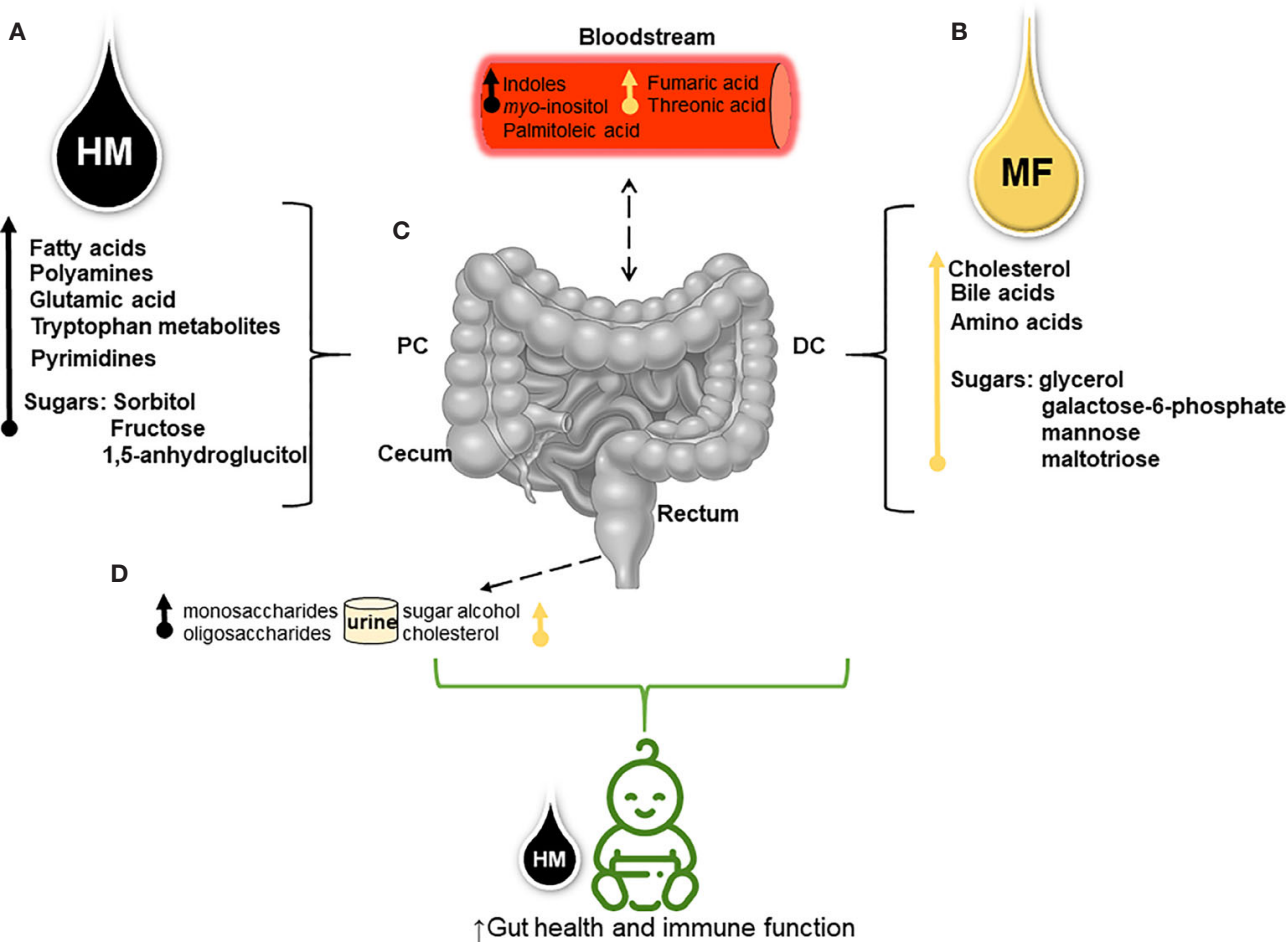
Schematic overview shows the divergent metabolite profile derived from human milk (HM) and dairy-based milk-formula (MF) and their potential effects on neonates’ intestinal metabolism **(A)**. Through metabolomics analysis higher fatty acids (myristic, palmitic, linolenic, linoleic, oleic, and palmitoleic acids), spermidine (polyamine), the glutamic amino acid, tryptophan and its derivatives, pyrimidines (thymine, pseudo-uridine, and uracil), and carbohydrates (sugars) were detected in different regions of the distal gastrointestinal tract (gut) [lumen of cecum, proximal colon (PC), distal colon (DC), and rectum] of HM-fed piglets **(B)**. While cholesterol abundance, bile acids (chenodeoxycholic and deoxycholic), essential amino acids (histidine, valine, and leucine), non-essential amino acids (taurine and glycine), and carbohydrates were greater in the luminal distal gut of MF- fed piglets during the first 21 days of life **(C)**. Sugar metabolites and tryptophan derivatives (i.e., indoles) present in the distal gut suggest that neonatal diet interactions with the host-microbiota impact the intestinal metabolism which can be associated with the altered serum metabolites from both diets **(D)**. Diet- microbial interactions reflected in the excretion of mono- and oligosaccharides (i.e., 1,5-anhydroglucitol and raffinose, respectively) in the urine of HM-group compared to sugar alcohols (i.e., threitol) and cholesterol abundance in the urine of MF-group. This model suggests that both HM and MF can impact the host-microbial and the host-intermediate metabolism resulting in a different metabolic profile prior to weaning.

Overall, our results showed a distinct metabolome signature between HM and MF-fed during the first 21 days of life. The data presented at PND 21 suggest that human milk feeding may favor the fatty acid metabolism for energy source while MF feeding utilized the sugar breakdown as fuel which is similar with the findings in breastfed vs formula fed infants (11, 13). The greater polyamines and tryptophan pathway metabolites within the distal gut of the HM-fed group may indicate a robust immune response upon human milk than with formula feeding. Also, at PND 21 the higher cholesterol and bile acids in the distal gut of the MF-fed piglets relative to the HM group suggests an impact of formula on cholesterol homeostasis. In contrast, the addition of complementary food (PND 51) resulted in a metabolite profile not as distinguishable and likely shifted to carbohydrate metabolism in HM group. Thus, diet and host-microbiota interactions likely played a role in luminal metabolome (**Figure 3**). Future studies are needed to determine how host physiology (liver and gut tissue) and immune system are impacted at the molecular level by post-weaning neonatal diet.

## Data Availability Statement

The raw metabolite data are available online as **Supplementary Table 6**. Further inquiries can be directed to the corresponding author.

## Ethics Statement

The animal study was reviewed and approved by University of Arkansas For Medical Sciences.

## Author Contributions

LY - conceived the study. FR and LY - conducted data anlyses and interpretation, and wrote the manuscript. KM and AB—conducted the study. KW—statistical analysis of the data, AE—input on data analysis, SA and LB—edited the manuscript. All authors contributed to the article and approved the submitted version.

## Funding

The project is funded by USDA-ARS Project 6026-51000-012-05S and 6026-51000-012-06S, and LY is also supported by NIH 1R21AI146521.

## Conflict of Interest

The authors declare that the article was conducted in the absence of any commercial or financial relationships that could be construed as a potential conflict of interest.

## Supplementary Material

Supplementary Figure 1 Two-dimensional scores plot of partial square discriminant analysis (PLS-DA) model showing the distribution of the luminal contents used as quality control pools in the metabolomic analysis. PLS-DA scores (i.e., individual samples) for components 1 and 2 are displayed. Gray circle shadow represents the 95% confidence region. Red circles indicate the individual luminal content samples.

Supplementary Table 1 Prior to metabolome data statistical analysis at PND 51, metabolite abundance in cecum, proximal colon, distal colon, and rectum contents were assessed by permutational multivariate ANOVA (PERMANOVA) including Diet (human milk or milk formula), group (immunization vs control), and their interactions (Diet:group).

Supplementary Table 2 Average abundances (quantifier ion [quantion] intensities) of metabolites significantly altered by diet at postnatal day (PND) 21 (n=8-11/group) across the cecum, proximal colon, distal colon, and rectum contents of piglets fed with human milk (HM) or milk formula (MF) through PND 21.

Supplementary Table 3 Average abundances (quantifier ion [quantion] intensities) of metabolites significantly altered by diet at postnatal day (PND) 51 (n=9–15/group) across the cecum, proximal colon, distal colon, and rectum contents of piglets fed with human milk (HM) or milk formula (MF) through PND 21.

Supplementary Table 4 Average abundances (quantifier ion [quantion] intensities) of serum metabolites significantly altered by diet at postnatal day (PND) 21 (n=25/group) and PND 51 (n=15/group) of piglets fed with human milk (HM) or milk formula (MF) through PND 21.

Supplementary Table 5 Average abundances (quantion peak intensities) of urinary metabolites significantly altered by diet at postnatal day (PND) 21 (n=25/group) and PND 51 (n=15/group), in piglets fed with human milk (HM) or milk formula (MF) through PND 21.
