# Supplementary figures and images for: Neonatal Diet Impacts the Large Intestine Luminal Metabolome at Weaning and Post-Weaning in Piglets Fed Formula or Human Milk
Source: Front Immunol. 2020 Dec 7;11:607609. doi: 10.3389/fimmu.2020.607609 (PMC7750455; doi:10.3389/fimmu.2020.607609)

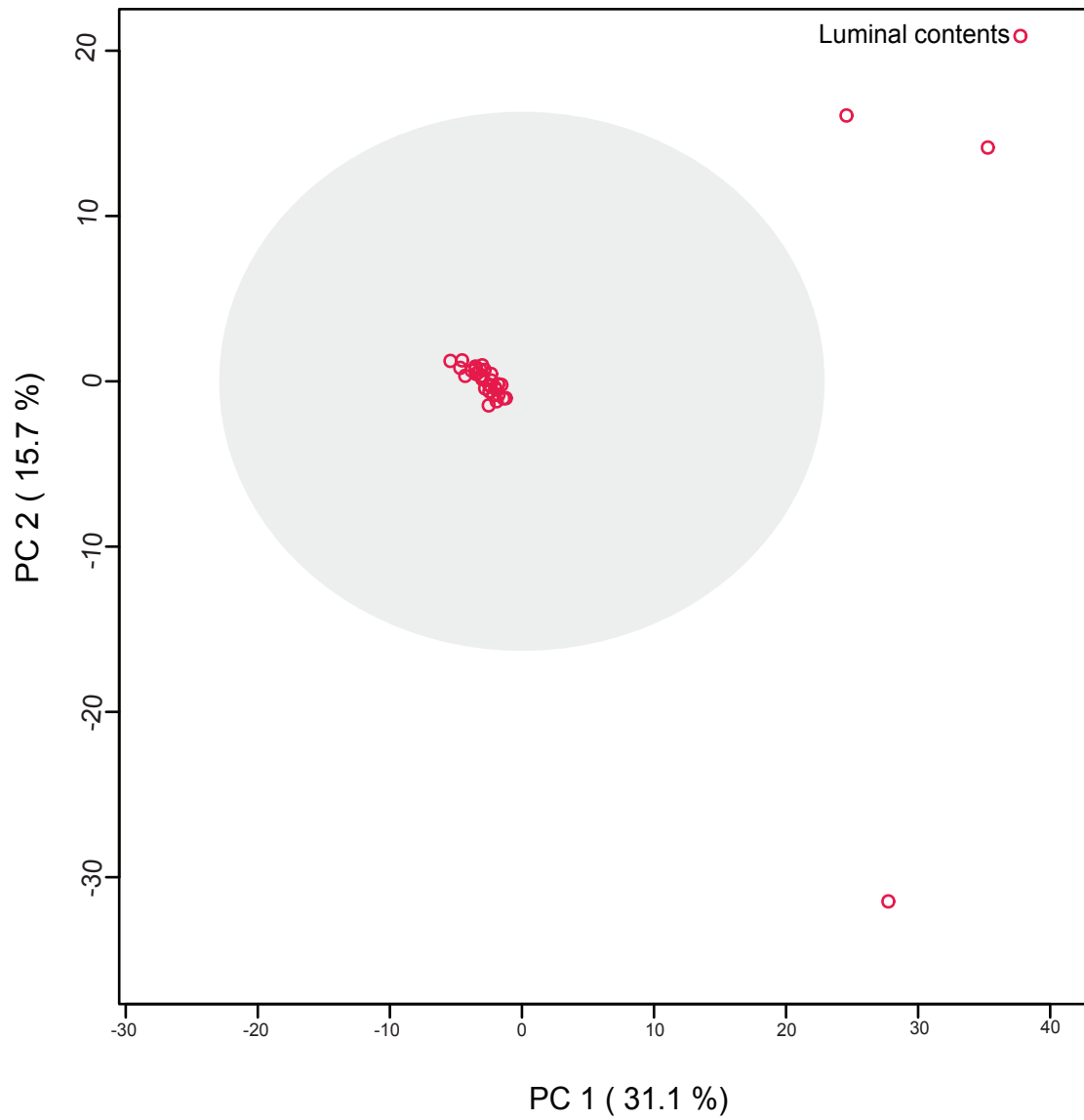

Supplement: Supplementary Figure 1 — Two-dimensional scores plot of partial square discriminant analysis (PLS-DA) model showing the distribution of the luminal contents used as quality control pools in the metabolomic analysis. PLS-DA scores (i.e., individual samples) for components 1 and 2 are displayed. Gray circle shadow represents the 95% confidence region. Red circles indicate the individual luminal content samples. [file Image_1.pdf]
