# Supplementary material for: Neonatal Diet Impacts the Large Intestine Luminal Metabolome at Weaning and Post-Weaning in Piglets Fed Formula or Human Milk
Source: Front Immunol. 2020 Dec 7;11:607609. doi: 10.3389/fimmu.2020.607609 (PMC7750455; doi:10.3389/fimmu.2020.607609)
Supplement: Supplementary Table 1 — Prior to metabolome data statistical analysis at PND 51, metabolite abundance in cecum, proximal colon, distal colon, and rectum contents were assessed by permutational multivariate ANOVA (PERMANOVA) including Diet (human milk or milk formula), group (immunization vs control), and their interactions (Diet:group). [file Table_1.docx]

| **Cecum** | **Degrees of Freedom** | **Sums of Sqs** | **F. Model** | ***P*r (> F)** |
| --- | --- | --- | --- | --- |
| Diet | 1 | 0.06 | 1.41 | 0.18 |
| Group | 1 | 0.10 | 2.51 | 0.03 |
| Diet:group | 1 | 0.05 | 1.27 | 0.25 |
| Residuals | 26 | 1.09 |  |  |
| **Proximal colon** | **Degrees of Freedom** | **Sums of Sqs** | **F. Model** | ***P*r (> F)** |
| Diet | 1 | 0.03 | 0.57 | 0.75 |
| Group | 1 | 0.03 | 0.65 | 0.65 |
| Diet:group | 1 | 0.04 | 0.90 | 0.42 |
| Residuals | 18 | 0.82 |  |  |
| **Distal colon** | **Degrees of Freedom** | **Sums of Sqs** | **F. Model** | ***P*r (> F)** |
| Diet | 1 | 0.03 | 1.66 | 0.15 |
| Group | 1 | 0.01 | 0.70 | 0.57 |
| Diet:group | 1 | 0.02 | 0.85 | 0.46 |
| Residuals | 26 | 0.56 |  |  |
| **Rectum** | **Degrees of Freedom** | **Sums of Sqs** | **F. Model** | ***P*r (> F)** |
| Diet | 1 | 0.03 | 1.38 | 0.22 |
| Group | 1 | 0.007 | 0.35 | 0.92 |
| Diet:group | 1 | 0.04 | 1.95 | 0.11 |
| Residuals | 20 | 0.40 |  |  |
