# Supplementary material for: Neonatal Diet Impacts the Large Intestine Luminal Metabolome at Weaning and Post-Weaning in Piglets Fed Formula or Human Milk
Source: Front Immunol. 2020 Dec 7;11:607609. doi: 10.3389/fimmu.2020.607609 (PMC7750455; doi:10.3389/fimmu.2020.607609)
Supplement: Supplementary Table 2 — Average abundances (quantifier ion [quantion] intensities) of metabolites significantly altered by diet at postnatal day (PND) 21 (n=8-11/group) across the cecum, proximal colon, distal colon, and rectum contents of piglets fed with human milk (HM) or milk formula (MF) through PND 21. [file Table_2.docx]

Average abundances (quantifier ion [quantion] intensities) of metabolites significantly different when comparing human milk (HM) or milk formula (MF) diet groups, in cecum, proximal colon, distal colon, and rectum contents of piglets at postnatal day (PND) 21.

| **Cecum** | **HM^1^** | **SEM^2^** | **MF^1^** | **SEM^2^** | **FC^3^** | **P^4^** | **FDR^5^** | **VIP^6^** |
| --- | --- | --- | --- | --- | --- | --- | --- | --- |
| delta-tocopherol | 1738 | 204 | 63258 | 6973 | 0.03 | < 0.01 | < 0.01 | 2.08 |
| gamma-tocopherol | 8553 | 1146 | 261087 | 19688 | 0.03 | < 0.01 | < 0.01 | 2.07 |
| beta-tocopherol | 1488 | 223 | 9937 | 735 | 0.15 | < 0.01 | < 0.01 | 1.98 |
| 1-methylinosine | 122 | 20 | 582 | 35 | 0.21 | < 0.01 | < 0.01 | 1.96 |
| 2,8-dihydroxyquinoline | 103 | 16 | 1726 | 374 | 0.06 | < 0.01 | < 0.01 | 1.92 |
| melezitose | 202 | 32 | 696 | 88 | 0.29 | < 0.01 | < 0.01 | 1.82 |
| behenic acid | 62790 | 5892 | 152628 | 8978 | 0.41 | < 0.01 | < 0.01 | 1.80 |
| cholesterol | 8019 | 1200 | 30126 | 3223 | 0.27 | < 0.01 | < 0.01 | 1.79 |
| erythronic acid lactone | 138 | 21 | 410 | 39 | 0.34 | < 0.01 | < 0.01 | 1.77 |
| 4-pyridoxic acid | 187 | 25 | 867 | 112 | 0.22 | < 0.01 | < 0.01 | 1.76 |
| 3,6-anhydro-D-galactose | 187 | 36 | 1658 | 354 | 0.11 | < 0.01 | < 0.01 | 1.69 |
| UDP-glucuronic acid | 897 | 244 | 3465 | 471 | 0.26 | < 0.01 | < 0.01 | 1.67 |
| 3-hydroxyphenylacetic acid | 620 | 67 | 1421 | 159 | 0.44 | < 0.01 | < 0.01 | 1.65 |
| lignoceric acid | 12592 | 1279 | 28137 | 2057 | 0.45 | < 0.01 | < 0.01 | 1.65 |
| dihydro-3-coumaric acid | 726 | 89 | 1852 | 231 | 0.39 | < 0.01 | < 0.01 | 1.65 |
| acetophenone | 21901 | 1643 | 38277 | 2820 | 0.57 | < 0.01 | < 0.01 | 1.64 |
| butyrolactam | 1833 | 279 | 3924 | 359 | 0.47 | < 0.01 | < 0.01 | 1.62 |
| deoxycholic acid | 1040 | 193 | 7030 | 1706 | 0.15 | < 0.01 | < 0.01 | 1.62 |
| palmitoleic acid | 1581 | 214 | 604 | 89 | 2.62 | < 0.01 | < 0.01 | 1.60 |
| glycerol-3-galactoside | 708 | 94 | 1912 | 344 | 0.37 | < 0.01 | < 0.01 | 1.60 |
| 2,5-dihydroxypyrazine | 308 | 50 | 683 | 65 | 0.45 | < 0.01 | < 0.01 | 1.57 |
| threonic acid | 1056 | 268 | 3208 | 386 | 0.33 | < 0.01 | < 0.01 | 1.56 |
| glutaric acid | 1605 | 396 | 4663 | 530 | 0.34 | < 0.01 | < 0.01 | 1.54 |
| 2-monopalmitin | 13045 | 4085 | 43232 | 3084 | 0.30 | < 0.01 | < 0.01 | 1.53 |
| 3-hydroxyanthranilic acid | 122 | 20 | 407 | 81 | 0.30 | < 0.01 | < 0.01 | 1.52 |
| ethanolamine | 9217 | 939 | 21564 | 2952 | 0.43 | < 0.01 | < 0.01 | 1.52 |
| maleimide | 5383 | 738 | 10573 | 1136 | 0.51 | < 0.01 | < 0.01 | 1.51 |
| hydroquinone | 630 | 77 | 1076 | 66 | 0.59 | < 0.01 | < 0.01 | 1.49 |
| norvaline | 1832 | 261 | 4169 | 496 | 0.44 | < 0.01 | < 0.01 | 1.49 |
| glucose-1-phosphate | 1059 | 240 | 2373 | 262 | 0.45 | < 0.01 | < 0.01 | 1.48 |
| enterolactone | 203 | 46 | 465 | 59 | 0.44 | < 0.01 | < 0.01 | 1.46 |
| galactose-6-phosphate | 82 | 11 | 216 | 39 | 0.38 | < 0.01 | < 0.01 | 1.46 |
| 4-aminobenzoicacid | 172 | 17 | 333 | 37 | 0.52 | < 0.01 | 0.01 | 1.45 |
| glycyl tyrosine | 488 | 60 | 963 | 75 | 0.51 | < 0.01 | 0.01 | 1.44 |
| histidine | 2041 | 607 | 4831 | 639 | 0.42 | < 0.01 | 0.01 | 1.43 |
| alpha-aminoadipic acid | 534 | 84 | 1655 | 308 | 0.32 | < 0.01 | 0.01 | 1.43 |
| linoleic acid | 9148 | 1503 | 3055 | 604 | 2.99 | < 0.01 | 0.01 | 1.43 |
| 2-deoxytetronic acid | 829 | 143 | 2909 | 580 | 0.28 | < 0.01 | 0.01 | 1.42 |
| valine | 47321 | 11157 | 121492 | 16114 | 0.39 | < 0.01 | 0.01 | 1.42 |
| beta sitosterol | 23953 | 10533 | 196896 | 50375 | 0.12 | < 0.01 | 0.01 | 1.42 |
| p-cresol | 1639 | 181 | 2612 | 191 | 0.63 | < 0.01 | 0.01 | 1.41 |
| cysteine | 832 | 135 | 2285 | 382 | 0.36 | < 0.01 | 0.01 | 1.40 |
| lyxose | 823 | 266 | 1902 | 227 | 0.43 | < 0.01 | 0.01 | 1.40 |
| digalacturonic acid | 200 | 32 | 454 | 68 | 0.44 | < 0.01 | 0.01 | 1.38 |
| N-acetyl-D-tryptophan | 239 | 84 | 1231 | 284 | 0.19 | < 0.01 | 0.01 | 1.38 |
| 3-hydroxypropionic acid | 3763 | 607 | 6789 | 634 | 0.55 | < 0.01 | 0.01 | 1.37 |
| isomaltose | 428 | 59 | 717 | 60 | 0.60 | < 0.01 | 0.01 | 1.37 |
| adipic acid | 4382 | 737 | 9898 | 1550 | 0.44 | < 0.01 | 0.01 | 1.36 |
| succinate semialdehyde | 1015 | 123 | 1661 | 139 | 0.61 | < 0.01 | 0.01 | 1.35 |
| erythrose | 193 | 40 | 395 | 53 | 0.49 | < 0.01 | 0.01 | 1.35 |
| p-hydroxylphenyllactic acid | 311 | 25 | 560 | 68 | 0.56 | < 0.01 | 0.01 | 1.34 |
| 2-monoolein | 3165 | 347 | 8020 | 1593 | 0.39 | < 0.01 | 0.01 | 1.33 |
| 5,6-dihydrouracil | 351 | 48 | 615 | 61 | 0.57 | < 0.01 | 0.01 | 1.33 |
| butylamine | 2538 | 352 | 3996 | 315 | 0.64 | < 0.01 | 0.01 | 1.31 |
| oleic acid | 58553 | 22709 | 7959 | 1415 | 7.36 | < 0.01 | 0.01 | 1.31 |
| indole-3-propionic acid | 2155 | 539 | 5569 | 989 | 0.39 | < 0.01 | 0.01 | 1.31 |
| taurine | 75 | 5 | 152 | 23 | 0.49 | < 0.01 | 0.01 | 1.31 |
| ribonic acid | 366 | 60 | 876 | 129 | 0.42 | < 0.01 | 0.01 | 1.31 |
| lyxitol | 1082 | 213 | 2273 | 342 | 0.48 | < 0.01 | 0.02 | 1.27 |
| salicylic acid | 638 | 155 | 1121 | 133 | 0.57 | < 0.01 | 0.02 | 1.27 |
| parabanic acid | 4242 | 549 | 8014 | 1087 | 0.53 | < 0.01 | 0.02 | 1.26 |
| 2-deoxypentitol | 113 | 20 | 209 | 25 | 0.54 | < 0.01 | 0.02 | 1.25 |
| linolenic acid | 16553 | 2620 | 6639 | 1319 | 2.49 | < 0.01 | 0.02 | 1.24 |
| orotic acid | 341 | 58 | 634 | 98 | 0.54 | < 0.01 | 0.02 | 1.24 |
| arachidic acid | 147053 | 15029 | 209967 | 12289 | 0.70 | 0.01 | 0.02 | 1.23 |
| cystathionine | 297 | 47 | 515 | 56 | 0.58 | 0.01 | 0.02 | 1.23 |
| 4-methylcatechol | 102 | 18 | 216 | 45 | 0.47 | 0.01 | 0.02 | 1.23 |
| palmitic acid | 625873 | 39506 | 444733 | 43594 | 1.41 | 0.01 | 0.02 | 1.23 |
| isoleucine | 39144 | 7645 | 81579 | 12819 | 0.48 | 0.01 | 0.02 | 1.22 |
| glucoheptulose | 384 | 75 | 1061 | 221 | 0.36 | 0.01 | 0.02 | 1.22 |
| 4-hydroxybutyric acid | 2879 | 533 | 4755 | 481 | 0.61 | 0.01 | 0.03 | 1.21 |
| pinitol | 119 | 20 | 222 | 38 | 0.54 | 0.01 | 0.03 | 1.21 |
| myristic acid | 145457 | 21036 | 73124 | 18704 | 1.99 | 0.01 | 0.03 | 1.20 |
| 3-phenyllactic acid | 226 | 32 | 452 | 75 | 0.50 | 0.01 | 0.03 | 1.19 |
| O-phosphoserine | 83 | 18 | 137 | 14 | 0.60 | 0.01 | 0.03 | 1.18 |
| allantoic acid | 510 | 103 | 1408 | 368 | 0.36 | 0.01 | 0.03 | 1.18 |
| dihydroxymalonic acid | 79 | 17 | 130 | 14 | 0.61 | 0.01 | 0.03 | 1.17 |
| lactobionic acid | 389 | 111 | 1007 | 290 | 0.39 | 0.01 | 0.03 | 1.17 |
| dihydroxyacetone | 9180 | 1648 | 15606 | 1865 | 0.59 | 0.01 | 0.03 | 1.17 |
| threitol | 585 | 126 | 992 | 137 | 0.59 | 0.01 | 0.03 | 1.17 |
| serotonin | 403 | 35 | 857 | 221 | 0.47 | 0.01 | 0.03 | 1.16 |
| glutamine | 61285 | 7762 | 88164 | 6917 | 0.70 | 0.01 | 0.04 | 1.15 |
| phenylacetic acid | 10284 | 3114 | 30418 | 9257 | 0.34 | 0.01 | 0.04 | 1.13 |
| citrulline | 2508 | 320 | 4751 | 810 | 0.53 | 0.01 | 0.04 | 1.13 |
| 3-3-hydroxyphenylpropionic acid | 726 | 89 | 1535 | 270 | 0.47 | 0.01 | 0.04 | 1.13 |
| propane-1,3-diol | 5522 | 758 | 8106 | 715 | 0.68 | 0.01 | 0.04 | 1.13 |
| 5-aminovaleric acid | 32622 | 20088 | 51457 | 8800 | 0.63 | 0.01 | 0.04 | 1.13 |
| pyrrole-2-carboxylic acid | 849 | 144 | 1424 | 169 | 0.60 | 0.01 | 0.04 | 1.12 |
| leucine | 68267 | 14347 | 118450 | 16248 | 0.58 | 0.01 | 0.04 | 1.12 |
| methionine | 6886 | 1252 | 11264 | 1274 | 0.61 | 0.01 | 0.04 | 1.12 |
| aconitic acid | 93 | 14 | 186 | 39 | 0.50 | 0.01 | 0.04 | 1.11 |
| tetracosane | 1132 | 135 | 1523 | 107 | 0.74 | 0.01 | 0.04 | 1.11 |
| phthalic acid | 10598 | 2095 | 20476 | 2761 | 0.52 | 0.01 | 0.04 | 1.11 |
| isolinoleic acid | 679 | 140 | 1140 | 137 | 0.60 | 0.01 | 0.04 | 1.10 |
| 3-hydroxybenzoic acid | 672 | 109 | 1049 | 123 | 0.64 | 0.02 | 0.04 | 1.10 |
| lactic acid | 28227 | 4532 | 68400 | 15592 | 0.41 | 0.02 | 0.04 | 1.10 |
| phytosphingosine | 404 | 94 | 1751 | 888 | 0.23 | 0.02 | 0.04 | 1.10 |
| glycerol | 231576 | 20963 | 340232 | 34945 | 0.68 | 0.02 | 0.05 | 1.08 |
| cytosin | 529 | 101 | 879 | 125 | 0.60 | 0.02 | 0.05 | 1.08 |
| maltotriose | 356 | 81 | 1456 | 515 | 0.24 | 0.02 | 0.05 | 1.08 |
| ribitol | 1465 | 195 | 2561 | 325 | 0.57 | 0.02 | 0.05 | 1.07 |
| N-acetylglycine | 523 | 64 | 789 | 92 | 0.66 | 0.02 | 0.05 | 1.06 |
| 3-phosphoglycerate | 158 | 21 | 226 | 20 | 0.70 | 0.02 | 0.06 | 1.06 |
| octadecanol | 10377 | 1439 | 15613 | 1935 | 0.66 | 0.02 | 0.06 | 1.05 |
| cystine | 421 | 70 | 639 | 91 | 0.66 | 0.02 | 0.06 | 1.05 |
| ferulic acid | 101 | 19 | 178 | 34 | 0.57 | 0.02 | 0.06 | 1.05 |
| squalene | 5731 | 1290 | 18650 | 4363 | 0.31 | 0.02 | 0.06 | 1.05 |
| homoserine | 844 | 143 | 1778 | 327 | 0.47 | 0.02 | 0.06 | 1.05 |
| 3-hydroxybutyric acid | 6327 | 1024 | 10901 | 1014 | 0.58 | 0.02 | 0.06 | 1.05 |
| 2,6-diaminopimelic acid | 206 | 42 | 345 | 48 | 0.60 | 0.02 | 0.06 | 1.04 |
| stearic acid | 4829607 | 212505 | 3878986 | 321597 | 1.25 | 0.03 | 0.06 | 1.03 |
| hydrocinnamic acid | 2436 | 703 | 30640 | 24729 | 0.08 | 0.03 | 0.06 | 1.02 |
| 3-hydroxy-3-methylglutaric acid | 103 | 19 | 146 | 12 | 0.71 | 0.03 | 0.06 | 1.02 |
| 4-hydroxybenzoic acid | 1081 | 199 | 1595 | 157 | 0.68 | 0.03 | 0.07 | 1.02 |
| methylmaleic acid | 135 | 21 | 191 | 15 | 0.71 | 0.03 | 0.07 | 1.01 |
| putrescine | 4460 | 3457 | 5720 | 1288 | 0.78 | 0.03 | 0.07 | 1.01 |
| raffinose | 157 | 34 | 328 | 95 | 0.48 | 0.03 | 0.07 | 1.01 |
| UDP-N-acetylglucosamine | 174 | 48 | 308 | 46 | 0.56 | 0.03 | 0.07 | 1.01 |
| beta-alanine | 2299 | 381 | 4117 | 514 | 0.56 | 0.03 | 0.07 | 1.01 |
| oxalic acid | 2139 | 414 | 4545 | 592 | 0.47 | 0.03 | 0.07 | 1.01 |
| glutamic acid | 611642 | 67690 | 383277 | 44281 | 1.60 | 0.03 | 0.07 | 1.00 |
| pyruvic acid | 15231 | 2354 | 77545 | 53319 | 0.20 | 0.03 | 0.07 | 1.00 |
| methionine sulfoxide | 6784 | 1389 | 11509 | 1964 | 0.59 | 0.03 | 0.07 | 1.00 |
| Proximal Colon |  |  |  |  |  |  |  |  |
| delta-tocopherol | 2979 | 519 | 74413 | 10318 | 0.04 | < 0.01 | < 0.01 | 2.26 |
| gamma-tocopherol | 13579 | 2016 | 269567 | 34241 | 0.05 | < 0.01 | < 0.01 | 2.25 |
| 2,5-dihydroxypyrazine | 634 | 27 | 1382 | 106 | 0.46 | < 0.01 | < 0.01 | 2.10 |
| 3,6-anhydro-D-galactose | 368 | 58 | 2040 | 288 | 0.18 | < 0.01 | < 0.01 | 2.08 |
| 1-methylinosine | 145 | 22 | 588 | 66 | 0.25 | < 0.01 | < 0.01 | 2.06 |
| tocopherol beta | 1833 | 219 | 10077 | 1419 | 0.18 | < 0.01 | < 0.01 | 2.05 |
| glycyl tyrosine | 846 | 56 | 1892 | 206 | 0.45 | < 0.01 | < 0.01 | 1.98 |
| UDP-glucuronic acid | 1068 | 177 | 4298 | 575 | 0.25 | < 0.01 | < 0.01 | 1.96 |
| palmitoleic acid | 4320 | 1317 | 785 | 112 | 5.51 | < 0.01 | < 0.01 | 1.91 |
| 3-phenyllactic acid | 341 | 51 | 867 | 91 | 0.39 | < 0.01 | < 0.01 | 1.89 |
| cholesterol | 6901 | 883 | 23671 | 2835 | 0.29 | < 0.01 | < 0.01 | 1.88 |
| erythronic acid lactone | 213 | 31 | 547 | 77 | 0.39 | < 0.01 | < 0.01 | 1.83 |
| 4-pyridoxic acid | 365 | 41 | 1319 | 179 | 0.28 | < 0.01 | < 0.01 | 1.83 |
| behenic acid | 70875 | 6344 | 157440 | 15665 | 0.45 | < 0.01 | < 0.01 | 1.83 |
| 2-ketoisocaproic acid | 9759 | 1230 | 34942 | 8500 | 0.28 | < 0.01 | < 0.01 | 1.82 |
| 2,8-dihydroxyquinoline | 255 | 35 | 703 | 85 | 0.36 | < 0.01 | < 0.01 | 1.80 |
| urea | 17990 | 994 | 34599 | 3915 | 0.52 | < 0.01 | < 0.01 | 1.78 |
| butyrolactam | 2295 | 208 | 4592 | 472 | 0.50 | < 0.01 | < 0.01 | 1.77 |
| oxalic acid | 2553 | 438 | 7389 | 1141 | 0.35 | < 0.01 | < 0.01 | 1.76 |
| 1-methyladenosine | 110 | 11 | 205 | 19 | 0.53 | < 0.01 | < 0.01 | 1.73 |
| digalacturonic acid | 213 | 30 | 438 | 48 | 0.49 | < 0.01 | < 0.01 | 1.71 |
| lyxitol | 1896 | 169 | 4041 | 506 | 0.47 | < 0.01 | < 0.01 | 1.70 |
| cysteine | 3074 | 561 | 7987 | 1215 | 0.38 | < 0.01 | < 0.01 | 1.70 |
| melezitose | 189 | 22 | 533 | 80 | 0.35 | < 0.01 | < 0.01 | 1.69 |
| alpha-aminoadipic acid | 1231 | 244 | 3708 | 585 | 0.33 | < 0.01 | < 0.01 | 1.69 |
| 5,6-dihydrouracil | 664 | 44 | 1064 | 88 | 0.62 | < 0.01 | < 0.01 | 1.66 |
| squalene | 7389 | 1368 | 28259 | 5168 | 0.26 | < 0.01 | 0.01 | 1.56 |
| myristic acid | 332535 | 78155 | 121609 | 23150 | 2.73 | < 0.01 | 0.01 | 1.55 |
| palmitic acid | 1127510 | 94825 | 752618 | 45928 | 1.50 | < 0.01 | 0.01 | 1.55 |
| 4-aminobenzoicacid | 295 | 39 | 825 | 176 | 0.36 | < 0.01 | 0.01 | 1.54 |
| 2-deoxytetronic acid | 1050 | 103 | 2257 | 455 | 0.47 | < 0.01 | 0.01 | 1.54 |
| norvaline | 2315 | 486 | 3978 | 267 | 0.58 | < 0.01 | 0.01 | 1.52 |
| 3-hydroxy-3-methylglutaric acid | 92 | 13 | 162 | 19 | 0.57 | < 0.01 | 0.01 | 1.51 |
| linoleic acid | 32011 | 5977 | 11235 | 2916 | 2.85 | < 0.01 | 0.01 | 1.51 |
| erythrose | 181 | 36 | 749 | 332 | 0.24 | < 0.01 | 0.01 | 1.49 |
| isopropylbenzene | 2026 | 196 | 3638 | 427 | 0.56 | < 0.01 | 0.01 | 1.48 |
| 2-monopalmitin | 2994 | 500 | 18574 | 5083 | 0.16 | < 0.01 | 0.01 | 1.47 |
| p-hydroxylphenyllactic acid | 475 | 42 | 795 | 92 | 0.60 | < 0.01 | 0.02 | 1.47 |
| dehydroascorbic acid | 9778 | 2168 | 4511 | 626 | 2.17 | < 0.01 | 0.02 | 1.46 |
| galactitol | 5648 | 2174 | 1427 | 613 | 3.96 | < 0.01 | 0.02 | 1.46 |
| tetracosane | 3854 | 380 | 5863 | 426 | 0.66 | < 0.01 | 0.02 | 1.45 |
| methanolphosphate | 941 | 123 | 2141 | 456 | 0.44 | < 0.01 | 0.02 | 1.45 |
| salicylaldehyde | 500 | 60 | 928 | 132 | 0.54 | < 0.01 | 0.02 | 1.44 |
| tartaric acid | 160 | 20 | 284 | 29 | 0.56 | < 0.01 | 0.02 | 1.43 |
| adipic acid | 5132 | 864 | 9322 | 1196 | 0.55 | < 0.01 | 0.02 | 1.41 |
| glycerol | 400598 | 34375 | 568545 | 43853 | 0.70 | < 0.01 | 0.02 | 1.40 |
| xylonolactone | 750 | 66 | 2162 | 544 | 0.35 | < 0.01 | 0.02 | 1.39 |
| 3-hydroxypropionic acid | 4866 | 641 | 7315 | 517 | 0.67 | < 0.01 | 0.02 | 1.39 |
| parabanic acid | 8233 | 1625 | 12461 | 1038 | 0.66 | < 0.01 | 0.03 | 1.37 |
| phosphate | 227793 | 36416 | 388201 | 41093 | 0.59 | < 0.01 | 0.03 | 1.37 |
| xanthosine | 353 | 46 | 516 | 31 | 0.69 | < 0.01 | 0.03 | 1.37 |
| isolinoleic acid | 1142 | 175 | 1887 | 139 | 0.61 | < 0.01 | 0.03 | 1.37 |
| propane-1,3-diol | 5734 | 711 | 8895 | 797 | 0.64 | < 0.01 | 0.03 | 1.36 |
| galactose-6-phosphate | 153 | 21 | 373 | 80 | 0.41 | 0.01 | 0.03 | 1.34 |
| lyxose | 1460 | 174 | 3064 | 548 | 0.48 | 0.01 | 0.03 | 1.34 |
| arachidic acid | 216361 | 19327 | 312631 | 25510 | 0.69 | 0.01 | 0.04 | 1.31 |
| N-acetylmannosamine | 9190 | 3731 | 28767 | 6822 | 0.32 | 0.01 | 0.04 | 1.31 |
| dodecanol | 2052 | 323 | 3092 | 283 | 0.66 | 0.01 | 0.04 | 1.30 |
| methylmaleic acid | 174 | 26 | 279 | 30 | 0.62 | 0.01 | 0.04 | 1.29 |
| allantoic acid | 692 | 92 | 1180 | 136 | 0.59 | 0.01 | 0.04 | 1.28 |
| butylamine | 1459 | 130 | 2287 | 258 | 0.64 | 0.01 | 0.04 | 1.28 |
| 3-hydroxyanthranilic acid | 163 | 23 | 321 | 71 | 0.51 | 0.01 | 0.04 | 1.28 |
| cellobiose | 2310 | 252 | 4098 | 613 | 0.56 | 0.01 | 0.05 | 1.26 |
| threitol | 629 | 62 | 946 | 105 | 0.67 | 0.01 | 0.05 | 1.25 |
| glutamic acid | 1176854 | 153757 | 697884 | 65464 | 1.69 | 0.01 | 0.05 | 1.25 |
| 3-aminoisobutyric acid | 2371 | 525 | 5210 | 897 | 0.46 | 0.01 | 0.05 | 1.24 |
| malonic acid | 686 | 86 | 1307 | 265 | 0.52 | 0.01 | 0.05 | 1.24 |
| 3-hydroxyphenylacetic acid | 884 | 137 | 1806 | 306 | 0.49 | 0.01 | 0.05 | 1.24 |
| threonic acid | 853 | 133 | 1771 | 218 | 0.48 | 0.01 | 0.05 | 1.24 |
| 3-3-hydroxyphenylpropionic acid | 1190 | 160 | 3440 | 1241 | 0.35 | 0.01 | 0.05 | 1.23 |
| p-cresol | 1766 | 154 | 2421 | 199 | 0.73 | 0.01 | 0.06 | 1.22 |
| triethanolamine | 886 | 85 | 1339 | 157 | 0.66 | 0.01 | 0.06 | 1.21 |
| 5-aminovaleric acid | 39929 | 14007 | 66744 | 8530 | 0.60 | 0.01 | 0.06 | 1.21 |
| 2-deoxypentitol | 113 | 10 | 182 | 25 | 0.62 | 0.02 | 0.06 | 1.21 |
| glutamine | 79147 | 9511 | 110157 | 9198 | 0.72 | 0.02 | 0.06 | 1.20 |
| citric acid | 10893 | 2521 | 7130 | 5561 | 1.53 | 0.02 | 0.06 | 1.20 |
| deoxycholic acid | 1570 | 393 | 4101 | 852 | 0.38 | 0.02 | 0.06 | 1.20 |
| linolenic acid | 32957 | 3956 | 17855 | 4297 | 1.85 | 0.02 | 0.06 | 1.20 |
| 4-hydroxybutyric acid | 3048 | 387 | 4512 | 520 | 0.68 | 0.02 | 0.06 | 1.19 |
| oleic acid | 159855 | 62469 | 39707 | 21273 | 4.03 | 0.02 | 0.06 | 1.18 |
| N-acetylaspartic acid | 24555 | 7547 | 12064 | 4117 | 2.04 | 0.02 | 0.07 | 1.16 |
| oleamide | 2384 | 608 | 4579 | 1345 | 0.52 | 0.02 | 0.07 | 1.16 |
| beta-alanine | 3330 | 451 | 5305 | 681 | 0.63 | 0.02 | 0.07 | 1.15 |
| pentitol | 113 | 9 | 153 | 13 | 0.74 | 0.02 | 0.07 | 1.15 |
| 2-methylglutaric acid | 703 | 218 | 1360 | 344 | 0.52 | 0.02 | 0.08 | 1.14 |
| chenodeoxycholic acid | 37595 | 13813 | 89407 | 29531 | 0.42 | 0.02 | 0.08 | 1.13 |
| dihydroxyacetone | 7431 | 946 | 10183 | 970 | 0.73 | 0.02 | 0.08 | 1.13 |
| lignoceric acid | 14160 | 1839 | 22982 | 2502 | 0.62 | 0.02 | 0.08 | 1.13 |
| raffinose | 180 | 28 | 303 | 42 | 0.60 | 0.02 | 0.08 | 1.13 |
| glycerol-3-galactoside | 2135 | 214 | 2967 | 294 | 0.72 | 0.03 | 0.08 | 1.12 |
| phytosphingosine | 855 | 88 | 1685 | 389 | 0.51 | 0.03 | 0.08 | 1.12 |
| aminomalonate | 1739 | 300 | 2559 | 280 | 0.68 | 0.03 | 0.08 | 1.12 |
| N-acetylglycine | 1176 | 217 | 1656 | 133 | 0.71 | 0.03 | 0.08 | 1.11 |
| pyrrole-2-carboxylic acid | 1863 | 357 | 2605 | 226 | 0.72 | 0.03 | 0.08 | 1.11 |
| N-acetylornithine | 1295 | 171 | 2047 | 236 | 0.63 | 0.03 | 0.09 | 1.10 |
| alpha-ketoglutarate | 6129 | 1938 | 16717 | 5114 | 0.37 | 0.03 | 0.09 | 1.09 |
| 2-monoolein | 7603 | 2023 | 13437 | 2788 | 0.57 | 0.03 | 0.09 | 1.09 |
| cis-gondoic acid | 3097 | 327 | 2050 | 225 | 1.51 | 0.03 | 0.09 | 1.08 |
| oxoproline | 1082563 | 344378 | 529181 | 44690 | 2.05 | 0.03 | 0.09 | 1.08 |
| enterolactone | 235 | 34 | 369 | 49 | 0.64 | 0.03 | 0.10 | 1.07 |
| glutaric acid | 1495 | 369 | 2924 | 577 | 0.51 | 0.04 | 0.10 | 1.06 |
| 1-monopalmitin | 7969 | 1676 | 3476 | 735 | 2.29 | 0.04 | 0.10 | 1.06 |
| nicotinamide | 1358 | 381 | 689 | 112 | 1.97 | 0.04 | 0.10 | 1.06 |
| citrulline | 10890 | 1458 | 16336 | 2209 | 0.67 | 0.04 | 0.11 | 1.05 |
| glucoheptulose | 689 | 86 | 1629 | 606 | 0.42 | 0.04 | 0.11 | 1.04 |
| dihydroxymalonic acid | 109 | 25 | 184 | 36 | 0.59 | 0.04 | 0.11 | 1.03 |
| lactobionic acid | 694 | 119 | 1187 | 209 | 0.58 | 0.04 | 0.11 | 1.03 |
| ethanolamine | 33228 | 6753 | 46323 | 5141 | 0.72 | 0.04 | 0.11 | 1.03 |
| melibiose | 131 | 16 | 200 | 30 | 0.66 | 0.05 | 0.12 | 1.01 |
| 4-methylcatechol | 108 | 14 | 178 | 33 | 0.61 | 0.05 | 0.12 | 1.01 |
| homocystine | 1027 | 99 | 1413 | 183 | 0.73 | 0.05 | 0.12 | 1.00 |
| Distal colon |  |  |  |  |  |  |  |  |
| delta-tocopherol | 3331 | 208 | 146962 | 18447 | 0.02 | < 0.01 | 0.00 | 2.41 |
| gamma-tocopherol | 21039 | 1917 | 517272 | 61629 | 0.04 | < 0.01 | 0.00 | 2.39 |
| 3,6-anhydro-D-galactose | 259 | 30 | 1922 | 256 | 0.13 | < 0.01 | 0.00 | 2.30 |
| beta-tocopherol | 3519 | 441 | 19796 | 2369 | 0.18 | < 0.01 | 0.00 | 2.22 |
| palmitoleic acid | 6926 | 880 | 821 | 100 | 8.44 | < 0.01 | 0.00 | 2.21 |
| 4-pyridoxic acid | 383 | 61 | 1536 | 191 | 0.25 | < 0.01 | 0.00 | 2.04 |
| erythronic acid lactone | 219 | 16 | 493 | 44 | 0.44 | < 0.01 | 0.00 | 2.02 |
| oleic acid | 469449 | 50482 | 73856 | 23936 | 6.36 | < 0.01 | 0.00 | 2.00 |
| melezitose | 364 | 52 | 921 | 87 | 0.39 | < 0.01 | 0.00 | 1.96 |
| galactitol | 8608 | 3342 | 882 | 76 | 9.76 | < 0.01 | 0.00 | 1.96 |
| 2,5-dihydroxypyrazine | 649 | 39 | 1142 | 98 | 0.57 | < 0.01 | 0.00 | 1.90 |
| cholesterol | 18311 | 3627 | 49675 | 4448 | 0.37 | < 0.01 | 0.00 | 1.90 |
| linoleic acid | 49933 | 5617 | 13986 | 5520 | 3.57 | < 0.01 | 0.00 | 1.87 |
| 1-methylinosine | 172 | 26 | 875 | 136 | 0.20 | < 0.01 | 0.00 | 1.87 |
| myristic acid | 700211 | 64821 | 291343 | 48434 | 2.40 | < 0.01 | 0.00 | 1.87 |
| glutamic acid | 930473 | 150262 | 306803 | 36781 | 3.03 | < 0.01 | 0.00 | 1.86 |
| 4-aminobenzoicacid | 516 | 91 | 1828 | 299 | 0.28 | < 0.01 | 0.00 | 1.86 |
| threonic acid | 869 | 213 | 2538 | 304 | 0.34 | < 0.01 | 0.00 | 1.86 |
| butyrolactam | 2684 | 329 | 4822 | 342 | 0.56 | < 0.01 | 0.00 | 1.84 |
| erythrose | 234 | 35 | 500 | 54 | 0.47 | < 0.01 | 0.00 | 1.80 |
| behenic acid | 131788 | 19242 | 337110 | 47168 | 0.39 | < 0.01 | 0.00 | 1.78 |
| glycyl tyrosine | 1107 | 145 | 2386 | 229 | 0.46 | < 0.01 | 0.00 | 1.78 |
| palmitic acid | 2023370 | 165035 | 1354469 | 78078 | 1.49 | < 0.01 | 0.00 | 1.73 |
| 2-deoxytetronic acid | 848 | 91 | 2282 | 479 | 0.37 | < 0.01 | 0.00 | 1.73 |
| pyrrole-2-carboxylic acid | 1033 | 120 | 1722 | 143 | 0.60 | < 0.01 | 0.01 | 1.70 |
| 5-hydroxy-3-indoleacetic acid | 776 | 80 | 344 | 77 | 2.25 | < 0.01 | 0.01 | 1.69 |
| oxoproline | 804959 | 135706 | 362926 | 23393 | 2.22 | < 0.01 | 0.01 | 1.69 |
| linolenic acid | 75731 | 14902 | 18827 | 4014 | 4.02 | < 0.01 | 0.01 | 1.68 |
| uracil | 66411 | 6435 | 37586 | 3383 | 1.77 | < 0.01 | 0.01 | 1.67 |
| glycerol-3-galactoside | 1620 | 229 | 3113 | 317 | 0.52 | < 0.01 | 0.01 | 1.67 |
| adipic acid | 4909 | 342 | 8866 | 1052 | 0.55 | < 0.01 | 0.01 | 1.65 |
| cis-gondoic acid | 5677 | 977 | 2122 | 345 | 2.68 | < 0.01 | 0.01 | 1.65 |
| deoxycholic acid | 2647 | 713 | 11300 | 1845 | 0.23 | < 0.01 | 0.01 | 1.63 |
| spermidine | 58259 | 7924 | 14837 | 7484 | 3.93 | < 0.01 | 0.01 | 1.62 |
| 2-methylglutaric acid | 792 | 361 | 3445 | 735 | 0.23 | < 0.01 | 0.01 | 1.62 |
| phenylacetic acid | 15123 | 1225 | 33074 | 4329 | 0.46 | < 0.01 | 0.01 | 1.61 |
| nicotinamide | 3295 | 712 | 1180 | 160 | 2.79 | < 0.01 | 0.01 | 1.61 |
| capric acid | 23579 | 2682 | 12097 | 1487 | 1.95 | < 0.01 | 0.01 | 1.56 |
| 1,5-anhydroglucitol | 2825 | 495 | 1337 | 156 | 2.11 | < 0.01 | 0.02 | 1.54 |
| galactose-6-phosphate | 136 | 22 | 354 | 80 | 0.38 | < 0.01 | 0.02 | 1.53 |
| ribonic acid | 292 | 35 | 777 | 163 | 0.38 | < 0.01 | 0.02 | 1.52 |
| trans-4-hydroxyproline | 28533 | 4362 | 15673 | 894 | 1.82 | < 0.01 | 0.02 | 1.51 |
| 1-monopalmitin | 15934 | 3189 | 3605 | 981 | 4.42 | < 0.01 | 0.02 | 1.51 |
| pseudo-uridine | 4903 | 664 | 2469 | 259 | 1.99 | < 0.01 | 0.02 | 1.49 |
| xylulose | 7403 | 984 | 3784 | 569 | 1.96 | < 0.01 | 0.02 | 1.49 |
| serotonin | 1445 | 359 | 2703 | 317 | 0.53 | < 0.01 | 0.03 | 1.47 |
| glutaric acid | 1120 | 238 | 3050 | 789 | 0.37 | < 0.01 | 0.03 | 1.45 |
| parabanic acid | 6945 | 1114 | 12013 | 1054 | 0.58 | 0.01 | 0.03 | 1.44 |
| p-cresol | 1865 | 139 | 2670 | 228 | 0.70 | 0.01 | 0.03 | 1.44 |
| ribose | 271496 | 42458 | 143274 | 20425 | 1.89 | 0.01 | 0.03 | 1.43 |
| hydroquinone | 791 | 81 | 1200 | 118 | 0.66 | 0.01 | 0.03 | 1.42 |
| 5,6-dihydrouracil | 670 | 55 | 948 | 70 | 0.71 | 0.01 | 0.04 | 1.40 |
| UDP-glucuronic acid | 1220 | 188 | 3145 | 637 | 0.39 | 0.01 | 0.04 | 1.39 |
| thymine | 13672 | 1954 | 7671 | 707 | 1.78 | 0.01 | 0.04 | 1.37 |
| lignoceric acid | 24263 | 4144 | 49436 | 9579 | 0.49 | 0.01 | 0.04 | 1.36 |
| arachidic acid | 403864 | 50734 | 648494 | 74183 | 0.62 | 0.01 | 0.05 | 1.35 |
| tryptophan | 24762 | 4056 | 13072 | 3373 | 1.89 | 0.01 | 0.05 | 1.35 |
| ornithine | 25204 | 3607 | 13324 | 2918 | 1.89 | 0.01 | 0.05 | 1.35 |
| N-acetylglutamate | 6120 | 706 | 3444 | 568 | 1.78 | 0.01 | 0.05 | 1.34 |
| panose | 423 | 65 | 884 | 160 | 0.48 | 0.01 | 0.05 | 1.34 |
| 3-hydroxypropionic acid | 5181 | 465 | 8146 | 967 | 0.64 | 0.01 | 0.05 | 1.33 |
| raffinose | 157 | 17 | 248 | 34 | 0.63 | 0.01 | 0.06 | 1.31 |
| cysteine | 1494 | 229 | 2757 | 403 | 0.54 | 0.01 | 0.06 | 1.30 |
| galactonic acid | 1524 | 295 | 835 | 78 | 1.82 | 0.01 | 0.06 | 1.29 |
| sorbitol | 12441 | 4608 | 3973 | 518 | 3.13 | 0.01 | 0.06 | 1.29 |
| uridine | 2344 | 323 | 4900 | 870 | 0.48 | 0.01 | 0.06 | 1.29 |
| N-acetylaspartic acid | 24116 | 10159 | 5426 | 869 | 4.44 | 0.02 | 0.07 | 1.27 |
| 4-hydroxybutyric acid | 3167 | 504 | 4498 | 347 | 0.70 | 0.02 | 0.07 | 1.25 |
| threitol | 621 | 102 | 945 | 94 | 0.66 | 0.02 | 0.08 | 1.24 |
| oxalic acid | 2206 | 336 | 3764 | 558 | 0.59 | 0.02 | 0.08 | 1.24 |
| cystine | 1026 | 90 | 759 | 75 | 1.35 | 0.02 | 0.08 | 1.23 |
| monomyristin | 3057 | 883 | 909 | 120 | 3.36 | 0.02 | 0.08 | 1.23 |
| squalene | 5889 | 1260 | 29695 | 10951 | 0.20 | 0.02 | 0.08 | 1.23 |
| 4-hydroxymandelic acid | 255 | 24 | 382 | 49 | 0.67 | 0.02 | 0.09 | 1.21 |
| dehydroascorbic acid | 20702 | 2122 | 11343 | 1560 | 1.83 | 0.02 | 0.09 | 1.20 |
| fructose | 8678 | 1031 | 5426 | 1139 | 1.60 | 0.03 | 0.10 | 1.19 |
| 3-hydroxybutyric acid | 7560 | 700 | 11569 | 1108 | 0.65 | 0.03 | 0.10 | 1.18 |
| aspartic acid | 93566 | 18584 | 49936 | 10028 | 1.87 | 0.03 | 0.10 | 1.18 |
| lactobionic acid | 682 | 144 | 989 | 87 | 0.69 | 0.03 | 0.11 | 1.15 |
| 2-monopalmitin | 41415 | 10533 | 12039 | 4682 | 3.44 | 0.03 | 0.12 | 1.14 |
| methylmaleic acid | 174 | 27 | 251 | 24 | 0.69 | 0.03 | 0.12 | 1.14 |
| hippuric acid | 1016 | 119 | 714 | 221 | 1.42 | 0.03 | 0.12 | 1.13 |
| propane-1,3-diol | 6994 | 695 | 10220 | 1092 | 0.68 | 0.04 | 0.13 | 1.12 |
| chenodeoxycholic acid | 33830 | 11018 | 82652 | 30280 | 0.41 | 0.04 | 0.13 | 1.12 |
| maltose | 4879 | 658 | 13195 | 3745 | 0.37 | 0.04 | 0.13 | 1.11 |
| pantothenic acid | 4397 | 1385 | 1508 | 318 | 2.92 | 0.04 | 0.13 | 1.10 |
| alpha-aminoadipic acid | 790 | 100 | 1745 | 337 | 0.45 | 0.04 | 0.14 | 1.10 |
| gluconic acid | 555 | 103 | 297 | 31 | 1.87 | 0.04 | 0.14 | 1.09 |
| lyxose | 1434 | 303 | 2334 | 462 | 0.61 | 0.04 | 0.14 | 1.08 |
| beta-alanine | 3706 | 953 | 6260 | 905 | 0.59 | 0.04 | 0.14 | 1.08 |
| UDP-N-acetylglucosamine | 259 | 48 | 372 | 43 | 0.70 | 0.05 | 0.15 | 1.07 |
| dihydroxyacetone | 8077 | 924 | 10676 | 880 | 0.76 | 0.05 | 0.15 | 1.07 |
| malonic acid | 766 | 53 | 1295 | 327 | 0.59 | 0.05 | 0.15 | 1.06 |
| beta sitosterol | 22094 | 6355 | 164750 | 62664 | 0.13 | 0.05 | 0.15 | 1.06 |
| stearic acid | 8584424 | 666445 | 10187891 | 291167 | 0.84 | 0.05 | 0.15 | 1.06 |
| Rectum |  |  |  |  |  |  |  |  |
| gamma-tocopherol | 22823 | 2647 | 583645 | 89870 | 0.04 | < 0.01 | < 0.01 | 2.64 |
| delta-tocopherol | 3283 | 568 | 175059 | 30650 | 0.02 | < 0.01 | < 0.01 | 2.61 |
| palmitoleic acid | 8349 | 1340 | 615 | 72 | 13.57 | < 0.01 | < 0.01 | 2.60 |
| threonic acid | 608 | 69 | 1709 | 215 | 0.36 | < 0.01 | < 0.01 | 2.29 |
| 1-methylinosine | 189 | 33 | 889 | 144 | 0.21 | < 0.01 | < 0.01 | 2.23 |
| beta-tocopherol | 4158 | 548 | 21803 | 3434 | 0.19 | < 0.01 | < 0.01 | 2.23 |
| 3,6-anhydro-D-galactose | 316 | 43 | 1160 | 194 | 0.27 | < 0.01 | < 0.01 | 2.19 |
| octadecanol | 48023 | 7704 | 102424 | 6513 | 0.47 | < 0.01 | < 0.01 | 2.05 |
| linoleic acid | 54138 | 8348 | 13835 | 3649 | 3.91 | < 0.01 | < 0.01 | 2.05 |
| 4-aminobenzoicacid | 960 | 215 | 2482 | 233 | 0.39 | < 0.01 | < 0.01 | 2.04 |
| 2,5-dihydroxypyrazine | 672 | 62 | 1227 | 119 | 0.55 | < 0.01 | < 0.01 | 2.00 |
| 4-pyridoxic acid | 415 | 99 | 1350 | 216 | 0.31 | < 0.01 | 0.01 | 1.99 |
| oleic acid | 440191 | 80906 | 85398 | 47031 | 5.15 | < 0.01 | 0.01 | 1.89 |
| glycyl tyrosine | 829 | 134 | 1909 | 158 | 0.43 | < 0.01 | 0.02 | 1.85 |
| glycine | 44615 | 3944 | 89099 | 11985 | 0.50 | < 0.01 | 0.02 | 1.85 |
| uric acid | 717 | 96 | 1719 | 275 | 0.42 | < 0.01 | 0.02 | 1.82 |
| oxalic acid | 2942 | 534 | 8202 | 1012 | 0.36 | < 0.01 | 0.02 | 1.80 |
| 5-hydroxy-3-indoleacetic acid | 824 | 87 | 429 | 83 | 1.92 | < 0.01 | 0.02 | 1.79 |
| palmitic acid | 1515125 | 64253 | 1102436 | 96584 | 1.37 | < 0.01 | 0.02 | 1.79 |
| 2,8-dihydroxyquinoline | 212 | 40 | 625 | 165 | 0.34 | < 0.01 | 0.03 | 1.76 |
| proline | 71923 | 9809 | 235145 | 56370 | 0.31 | < 0.01 | 0.03 | 1.75 |
| valine | 236629 | 26908 | 517077 | 87043 | 0.46 | < 0.01 | 0.03 | 1.74 |
| isolinoleic acid | 986 | 126 | 1815 | 220 | 0.54 | < 0.01 | 0.03 | 1.73 |
| alanine | 190573 | 39975 | 531593 | 95771 | 0.36 | < 0.01 | 0.03 | 1.70 |
| melezitose | 408 | 59 | 909 | 138 | 0.45 | < 0.01 | 0.03 | 1.70 |
| 2-deoxytetronic acid | 931 | 184 | 1906 | 288 | 0.49 | < 0.01 | 0.03 | 1.69 |
| deoxycholic acid | 2805 | 974 | 7852 | 1377 | 0.36 | < 0.01 | 0.04 | 1.68 |
| glutaric acid | 2312 | 565 | 6423 | 1507 | 0.36 | < 0.01 | 0.04 | 1.66 |
| isoleucine | 145147 | 17537 | 354847 | 68488 | 0.41 | < 0.01 | 0.04 | 1.66 |
| spermidine | 23474 | 6506 | 4243 | 3592 | 5.53 | < 0.01 | 0.04 | 1.65 |
| threonine | 30098 | 4222 | 70540 | 14278 | 0.43 | < 0.01 | 0.04 | 1.64 |
| 3-phenyllactic acid | 531 | 71 | 1217 | 206 | 0.44 | < 0.01 | 0.04 | 1.63 |
| leucine | 262738 | 27431 | 588107 | 113109 | 0.45 | 0.01 | 0.05 | 1.61 |
| 2-methylglutaric acid | 1743 | 560 | 8356 | 2548 | 0.21 | 0.01 | 0.05 | 1.60 |
| uridine | 2546 | 533 | 6115 | 1019 | 0.42 | 0.01 | 0.05 | 1.58 |
| linolenic acid | 67665 | 12297 | 21882 | 3716 | 3.09 | 0.01 | 0.06 | 1.57 |
| 2-hydroxybutanoic acid | 2918 | 427 | 5823 | 1021 | 0.50 | 0.01 | 0.06 | 1.56 |
| 2-ketoisocaproic acid | 10570 | 1503 | 21789 | 4002 | 0.49 | 0.01 | 0.06 | 1.55 |
| N-acetylaspartic acid | 10420 | 3025 | 3625 | 1075 | 2.87 | 0.01 | 0.06 | 1.55 |
| squalene | 7749 | 1536 | 33726 | 9789 | 0.23 | 0.01 | 0.07 | 1.52 |
| myristic acid | 632851 | 53966 | 401123 | 85521 | 1.58 | 0.01 | 0.07 | 1.51 |
| N-acetylglycine | 1260 | 180 | 2082 | 274 | 0.60 | 0.01 | 0.08 | 1.49 |
| methionine | 21104 | 3049 | 53916 | 13521 | 0.39 | 0.01 | 0.08 | 1.49 |
| phenylacetic acid | 29036 | 7652 | 66098 | 20305 | 0.44 | 0.01 | 0.08 | 1.47 |
| glutamic acid | 521372 | 106688 | 246722 | 34239 | 2.11 | 0.01 | 0.08 | 1.46 |
| nicotinamide | 867 | 179 | 352 | 42 | 2.46 | 0.01 | 0.09 | 1.45 |
| dehydroascorbic acid | 17685 | 1498 | 12059 | 2088 | 1.47 | 0.02 | 0.10 | 1.43 |
| ribonic acid | 395 | 50 | 658 | 99 | 0.60 | 0.02 | 0.10 | 1.42 |
| serine | 62009 | 4912 | 105634 | 16966 | 0.59 | 0.02 | 0.10 | 1.40 |
| methionine sulfoxide | 11897 | 2073 | 21463 | 3495 | 0.55 | 0.02 | 0.10 | 1.40 |
| maltotriose | 247 | 33 | 391 | 53 | 0.63 | 0.02 | 0.10 | 1.40 |
| mannose | 5318 | 867 | 9690 | 1390 | 0.55 | 0.02 | 0.10 | 1.40 |
| N-acetylornithine | 1798 | 312 | 974 | 238 | 1.85 | 0.02 | 0.11 | 1.38 |
| 1,5-anhydroglucitol | 2209 | 130 | 1674 | 224 | 1.32 | 0.02 | 0.12 | 1.36 |
| erythronic acid lactone | 277 | 57 | 397 | 41 | 0.70 | 0.03 | 0.13 | 1.34 |
| lactobionic acid | 555 | 58 | 1167 | 262 | 0.48 | 0.03 | 0.13 | 1.33 |
| phenylalanine | 48286 | 6454 | 108093 | 25076 | 0.45 | 0.03 | 0.13 | 1.33 |
| histidine | 6240 | 1424 | 14434 | 3220 | 0.43 | 0.03 | 0.14 | 1.32 |
| gluconic acid | 447 | 90 | 244 | 33 | 1.83 | 0.03 | 0.14 | 1.31 |
| nicotinic acid | 45153 | 12550 | 20726 | 3996 | 2.18 | 0.03 | 0.14 | 1.31 |
| Isoleucyl-Isoleucine | 666 | 106 | 968 | 93 | 0.69 | 0.03 | 0.14 | 1.30 |
| 3,4-dihydroxycinnamic acid | 703 | 136 | 1247 | 254 | 0.56 | 0.03 | 0.15 | 1.29 |
