# Supplementary material for: Neonatal Diet Impacts the Large Intestine Luminal Metabolome at Weaning and Post-Weaning in Piglets Fed Formula or Human Milk
Source: Front Immunol. 2020 Dec 7;11:607609. doi: 10.3389/fimmu.2020.607609 (PMC7750455; doi:10.3389/fimmu.2020.607609)
Supplement: Supplementary Table 3 — Average abundances (quantifier ion [quantion] intensities) of metabolites significantly altered by diet at postnatal day (PND) 51 (n=9–15/group) across the cecum, proximal colon, distal colon, and rectum contents of piglets fed with human milk (HM) or milk formula (MF) through PND 21. [file Table_3.docx]

Average abundances (quantifier ion [quantion] intensities) of metabolites significantly different when comparing human milk (HM) or milk formula (MF) diet groups, in cecum, proximal colon, distal colon, and rectum contents of piglets at postnatal day (PND) 51.

| **Cecum** | **HM^1^** | **SEM^2^** | **MF^1^** | **SEM^2^** | **FC^3^** | ***P*^4^** | **FDR^5^** | **VIP^6^** |
| --- | --- | --- | --- | --- | --- | --- | --- | --- |
| cysteine | 1047 | 86 | 1887 | 238 | 0.55 | < 0.01 | 0.17 | 3.06 |
| 3-hydroxyphenylacetic acid | 7833 | 1350 | 3209 | 876 | 2.44 | < 0.01 | 0.53 | 2.66 |
| pentadecanoic acid | 39636 | 4100 | 54316 | 3891 | 0.73 | 0.01 | 0.70 | 2.45 |
| 7-methylguanine | 164 | 15 | 361 | 94 | 0.45 | 0.01 | 0.70 | 2.36 |
| N-acetylornithine | 3134 | 702 | 1310 | 211 | 2.39 | 0.01 | 0.70 | 2.34 |
| phenaceturic acid | 3456 | 1400 | 11049 | 3089 | 0.31 | 0.02 | 0.78 | 2.14 |
| urocanic acid | 769 | 99 | 1167 | 162 | 0.66 | 0.02 | 0.78 | 2.13 |
| pantothenic acid | 491 | 49 | 689 | 71 | 0.71 | 0.03 | 0.78 | 2.12 |
| indole-3-propionic acid | 12716 | 2080 | 7397 | 1240 | 1.72 | 0.03 | 0.78 | 2.07 |
| erythritol | 1445 | 356 | 761 | 104 | 1.90 | 0.03 | 0.78 | 2.02 |
| orotic acid | 6114 | 3767 | 619 | 101 | 9.88 | 0.04 | 0.78 | 1.97 |
| glycyl tyrosine | 299 | 23 | 365 | 20 | 0.82 | 0.04 | 0.78 | 1.96 |
| 2-deoxytetronic acid | 7746 | 959 | 5503 | 786 | 1.41 | 0.05 | 0.78 | 1.90 |
| oleamide | 2150 | 212 | 1647 | 100 | 1.31 | 0.05 | 0.78 | 1.88 |
| levoinositol | 367 | 54 | 608 | 118 | 0.60 | 0.05 | 0.78 | 1.84 |
| Proximal colon |  |  |  |  |  |  |  |  |
| urocanic acid | 1553 | 151 | 2751 | 246 | 0.56 | < 0.01 | 0.05 | 3.14 |
| N-acetylaspartic acid | 15961 | 1962 | 8969 | 1068 | 1.78 | < 0.01 | 0.24 | 2.63 |
| lactitol | 1389 | 130 | 2460 | 309 | 0.56 | < 0.01 | 0.24 | 2.51 |
| homocystine | 1278 | 83 | 893 | 79 | 1.43 | < 0.01 | 0.24 | 2.51 |
| 3-hydroxyphenylacetic acid | 13295 | 1854 | 6209 | 1334 | 2.14 | < 0.01 | 0.24 | 2.50 |
| beta-glycerolphosphate | 1078 | 66 | 769 | 60 | 1.40 | 0.01 | 0.26 | 2.44 |
| 1-monostearin | 4694 | 923 | 10161 | 2304 | 0.46 | 0.02 | 0.68 | 2.11 |
| 4-hydroxyphenylacetic acid | 10240 | 2237 | 6079 | 992 | 1.68 | 0.02 | 0.68 | 2.10 |
| dehydroascorbic acid | 2174 | 226 | 2799 | 237 | 0.78 | 0.02 | 0.68 | 2.05 |
| glycerol-3-galactoside | 6029 | 857 | 9899 | 1364 | 0.61 | 0.03 | 0.68 | 2.00 |
| cystathionine | 8143 | 644 | 5922 | 746 | 1.38 | 0.03 | 0.68 | 1.96 |
| 3-hydroxypropionic acid | 10827 | 1347 | 7530 | 573 | 1.44 | 0.03 | 0.68 | 1.96 |
| N-acetylornithine | 5964 | 1444 | 2495 | 544 | 2.39 | 0.03 | 0.68 | 1.94 |
| propane-1,3-diol | 6955 | 478 | 5498 | 587 | 1.27 | 0.04 | 0.68 | 1.86 |
| UDP-glucuronic acid | 1141 | 373 | 1820 | 423 | 0.63 | 0.05 | 0.68 | 1.76 |
| Distal colon |  |  |  |  |  |  |  |  |
| dihydroxyacetone | 7091 | 281 | 9685 | 599 | 0.73 | < 0.01 | 0.08 | 2.66 |
| lyxose | 19364 | 3196 | 9660 | 950 | 2.00 | < 0.01 | 0.16 | 2.41 |
| xylitol | 2899 | 245 | 1950 | 118 | 1.49 | < 0.01 | 0.16 | 2.36 |
| xylose | 282684 | 50219 | 135380 | 17049 | 2.09 | < 0.01 | 0.16 | 2.25 |
| pentose | 74638 | 22946 | 27458 | 3058 | 2.72 | < 0.01 | 0.16 | 2.25 |
| thymidine-5-phosphate | 1519 | 586 | 413 | 63 | 3.68 | < 0.01 | 0.16 | 2.23 |
| tartaric acid | 190 | 21 | 279 | 22 | 0.68 | 0.01 | 0.22 | 2.11 |
| inosine | 1086 | 508 | 4642 | 1437 | 0.23 | 0.01 | 0.22 | 2.07 |
| putrescine | 5263 | 720 | 10496 | 1763 | 0.50 | 0.01 | 0.22 | 2.06 |
| uridine | 2481 | 266 | 8120 | 2164 | 0.31 | 0.01 | 0.22 | 2.03 |
| xylulose | 12922 | 1177 | 8456 | 1028 | 1.53 | 0.01 | 0.22 | 2.03 |
| pinitol | 276 | 53 | 1217 | 413 | 0.23 | 0.01 | 0.26 | 1.97 |
| butyrolactam | 4637 | 157 | 5324 | 203 | 0.87 | 0.02 | 0.30 | 1.89 |
| 3-hydroxyphenylacetic acid | 16716 | 2272 | 8953 | 1758 | 1.87 | 0.02 | 0.30 | 1.89 |
| behenic acid | 65712 | 3150 | 54919 | 3179 | 1.20 | 0.02 | 0.30 | 1.87 |
| monomyristin | 851 | 69 | 1200 | 134 | 0.71 | 0.02 | 0.30 | 1.86 |
| xanthine | 44628 | 4950 | 28212 | 5039 | 1.58 | 0.02 | 0.39 | 1.77 |
| uracil | 161340 | 19225 | 104156 | 12199 | 1.55 | 0.03 | 0.39 | 1.76 |
| ribose | 364271 | 36115 | 250569 | 34238 | 1.45 | 0.03 | 0.39 | 1.73 |
| thymine | 34509 | 8815 | 14749 | 2364 | 2.34 | 0.03 | 0.39 | 1.71 |
| sulfuric acid | 344 | 82 | 139 | 17 | 2.47 | 0.03 | 0.39 | 1.71 |
| beta-glycerolphosphate | 1565 | 135 | 1256 | 63 | 1.25 | 0.03 | 0.39 | 1.70 |
| cytosin | 7044 | 1669 | 3142 | 768 | 2.24 | 0.03 | 0.39 | 1.68 |
| quinolinic acid | 182 | 30 | 676 | 271 | 0.27 | 0.03 | 0.39 | 1.67 |
| pseudo-uridine | 11366 | 1550 | 7338 | 1227 | 1.55 | 0.04 | 0.39 | 1.64 |
| capric acid | 7461 | 607 | 10812 | 1365 | 0.69 | 0.04 | 0.39 | 1.62 |
| benzoic acid | 75600 | 3149 | 134444 | 22859 | 0.56 | 0.04 | 0.39 | 1.61 |
| gluconic acid lactone | 1044 | 199 | 634 | 131 | 1.65 | 0.05 | 0.39 | 1.58 |
| 2,8-dihydroxyquinoline | 11316 | 5093 | 95432 | 37785 | 0.12 | 0.05 | 0.39 | 1.58 |
| glycerol | 1083355 | 116997 | 811883 | 44275 | 1.33 | 0.05 | 0.39 | 1.58 |
| erythritol | 1116 | 255 | 652 | 40 | 1.71 | 0.05 | 0.39 | 1.57 |
| N-acetylornithine | 5690 | 1432 | 2656 | 400 | 2.14 | 0.05 | 0.39 | 1.57 |
| nicotinamide | 1093 | 65 | 1512 | 182 | 0.72 | 0.05 | 0.39 | 1.57 |
| cerotinic acid | 2136 | 254 | 2763 | 208 | 0.77 | 0.05 | 0.39 | 1.55 |
| 3-hydroxybutyric acid | 8278 | 1505 | 11853 | 1504 | 0.70 | 0.05 | 0.39 | 1.54 |
| 1,2-cyclohexanedione | 99332 | 9541 | 72770 | 9180 | 1.37 | 0.05 | 0.39 | 1.53 |
| phenylacetic acid | 50585 | 8327 | 73884 | 8921 | 0.68 | 0.05 | 0.39 | 1.53 |
| Rectum |  |  |  |  |  |  |  |  |
| erythritol | 655 | 32 | 432 | 41 | 1.52 | < 0.01 | 0.08 | 2.96 |
| 4-methylcatechol | 510 | 80 | 1502 | 367 | 0.34 | < 0.01 | 0.16 | 2.73 |
| maltotriose | 586 | 117 | 201 | 14 | 2.91 | < 0.01 | 0.30 | 2.53 |
| serotonin | 2124 | 200 | 3294 | 310 | 0.64 | 0.01 | 0.38 | 2.41 |
| glutamine | 62070 | 1987 | 53216 | 2734 | 1.17 | 0.01 | 0.59 | 2.23 |
| panose | 523 | 86 | 236 | 19 | 2.21 | 0.01 | 0.59 | 2.20 |
| creatine | 3216 | 353 | 8023 | 2272 | 0.40 | 0.02 | 0.63 | 2.10 |
| pinitol | 264 | 63 | 1606 | 687 | 0.16 | 0.02 | 0.63 | 2.07 |
| quinolinic acid | 153 | 29 | 1196 | 575 | 0.13 | 0.02 | 0.63 | 2.06 |
| methylmalonic acid | 45841 | 3067 | 35941 | 3215 | 1.28 | 0.03 | 0.65 | 1.97 |
| dihydro-3-coumaric acid | 18782 | 7156 | 254211 | 128593 | 0.07 | 0.03 | 0.65 | 1.94 |
| behenic acid | 40727 | 1532 | 35181 | 1820 | 1.16 | 0.03 | 0.65 | 1.92 |
| threitol | 553 | 26 | 466 | 27 | 1.19 | 0.03 | 0.65 | 1.92 |
| uridine | 2158 | 223 | 4097 | 909 | 0.53 | 0.03 | 0.65 | 1.91 |
| isomaltose | 706 | 116 | 445 | 40 | 1.59 | 0.03 | 0.65 | 1.90 |
| 3-3-hydroxyphenylpropionic acid | 20260 | 7225 | 271401 | 139542 | 0.07 | 0.04 | 0.65 | 1.87 |
| phenylacetic acid | 77171 | 12912 | 118377 | 17553 | 0.65 | 0.04 | 0.65 | 1.86 |
| ferulic acid | 1802 | 245 | 1189 | 355 | 1.52 | 0.05 | 0.67 | 1.80 |
| 2,8-dihydroxyquinoline | 9860 | 6040 | 138613 | 64438 | 0.07 | 0.05 | 0.67 | 1.79 |
| myristic acid | 28008 | 1691 | 34120 | 2512 | 0.82 | 0.05 | 0.67 | 1.78 |
| 3-hydroxypropionic acid | 9289 | 1478 | 5784 | 734 | 1.61 | 0.05 | 0.67 | 1.77 |
