# Supplementary material for: Neonatal Diet Impacts the Large Intestine Luminal Metabolome at Weaning and Post-Weaning in Piglets Fed Formula or Human Milk
Source: Front Immunol. 2020 Dec 7;11:607609. doi: 10.3389/fimmu.2020.607609 (PMC7750455; doi:10.3389/fimmu.2020.607609)
Supplement: Supplementary Table 5 — Average abundances (quantion peak intensities) of urinary metabolites significantly altered by diet at postnatal day (PND) 21 (n=25/group) and PND 51 (n=15/group), in piglets fed with human milk (HM) or milk formula (MF) through PND 21. [file Table_5.docx]

| **Urine PND 21** | **HM^1^** | **SEM^2^** | **MF^1^** | **SEM^2^** | **FC^3^** | ***P*^4^** | **FDR^5^** | **VIP^6^** |
| --- | --- | --- | --- | --- | --- | --- | --- | --- |
| xylonolactone | 3295 | 251 | 39710 | 2987 | 0.08 | < 0.01 | < 0.01 | 3.73 |
| tartaric acid | 8450 | 1182 | 180555 | 13579 | 0.05 | < 0.01 | < 0.01 | 3.65 |
| fucose | 1884181 | 283396 | 356826 | 35004 | 5.28 | < 0.01 | < 0.01 | 3.08 |
| threonic acid | 252261 | 23852 | 513055 | 31372 | 0.49 | < 0.01 | < 0.01 | 2.76 |
| raffinose | 2065 | 292 | 683 | 176 | 3.02 | < 0.01 | < 0.01 | 2.74 |
| beta-gentiobiose | 62366 | 6246 | 123309 | 9311 | 0.51 | < 0.01 | < 0.01 | 2.70 |
| beta sitosterol | 27815 | 5833 | 2414 | 386 | 11.52 | < 0.01 | < 0.01 | 2.70 |
| 2-monoolein | 12692 | 2480 | 72950 | 13584 | 0.17 | < 0.01 | < 0.01 | 2.66 |
| ribonic acid | 66882 | 5118 | 141502 | 11306 | 0.47 | < 0.01 | < 0.01 | 2.63 |
| glucose-1-phosphate | 79090 | 6201 | 168367 | 12995 | 0.47 | < 0.01 | < 0.01 | 2.55 |
| 2,8-dihydroxyquinoline | 5677 | 977 | 11039 | 850 | 0.51 | < 0.01 | < 0.01 | 2.40 |
| maltotriose | 2780 | 538 | 5954 | 616 | 0.47 | < 0.01 | < 0.01 | 2.36 |
| 6-hydroxynicotinic acid | 2346 | 346 | 5146 | 519 | 0.46 | < 0.01 | < 0.01 | 2.32 |
| pinitol | 3486 | 485 | 7176 | 825 | 0.49 | < 0.01 | < 0.01 | 2.12 |
| cholesterol | 843 | 88 | 1710 | 185 | 0.49 | < 0.01 | < 0.01 | 2.09 |
| 2-hydroxyvaleric acid | 370548 | 36110 | 199426 | 24448 | 1.86 | < 0.01 | < 0.01 | 2.05 |
| 4-pyridoxic acid | 1816 | 881 | 2384 | 231 | 0.76 | < 0.01 | < 0.01 | 2.00 |
| threitol | 185405 | 10665 | 261302 | 14255 | 0.71 | < 0.01 | < 0.01 | 1.99 |
| glutamine | 132117 | 10024 | 235132 | 31299 | 0.56 | < 0.01 | 0.01 | 1.88 |
| arachidonic acid | 3969 | 440 | 2204 | 250 | 1.80 | < 0.01 | 0.01 | 1.87 |
| erythritol | 1007123 | 61506 | 1372060 | 81916 | 0.73 | < 0.01 | 0.02 | 1.73 |
| glycolic acid | 111862 | 12054 | 70754 | 5664 | 1.58 | < 0.01 | 0.02 | 1.71 |
| 1-monostearin | 2054 | 169 | 3020 | 331 | 0.68 | < 0.01 | 0.02 | 1.66 |
| tocopherol beta | 6361 | 633 | 4083 | 798 | 1.56 | < 0.01 | 0.03 | 1.61 |
| urea | 1154860 | 338605 | 593584 | 311053 | 1.95 | < 0.01 | 0.04 | 1.58 |
| 1,5-anhydroglucitol | 87562 | 16481 | 33889 | 2871 | 2.58 | 0.01 | 0.05 | 1.51 |
| sucrose | 1298 | 340 | 4988 | 1720 | 0.26 | 0.01 | 0.05 | 1.50 |
| methylmalonic acid | 390084 | 47584 | 297960 | 110183 | 1.31 | 0.01 | 0.05 | 1.49 |
| gluconic acid lactone | 36145 | 3782 | 21400 | 3110 | 1.69 | 0.01 | 0.05 | 1.48 |
| cytosin | 10285 | 1093 | 6538 | 681 | 1.57 | 0.01 | 0.05 | 1.48 |
| phenylalanine | 42206 | 4868 | 78570 | 10286 | 0.54 | 0.01 | 0.05 | 1.48 |
| xanthurenic acid | 4011 | 387 | 2646 | 256 | 1.52 | 0.01 | 0.06 | 1.47 |
| homocystine | 1632 | 134 | 2365 | 287 | 0.69 | 0.01 | 0.11 | 1.36 |
| isopropylbenzene | 6686 | 740 | 4449 | 598 | 1.50 | 0.01 | 0.12 | 1.33 |
| cis-gondoic acid | 711 | 66 | 512 | 59 | 1.39 | 0.02 | 0.12 | 1.32 |
| adipic acid | 58881 | 8810 | 73217 | 5214 | 0.80 | 0.02 | 0.13 | 1.30 |
| cytidine | 2392 | 341 | 4653 | 1121 | 0.51 | 0.02 | 0.14 | 1.28 |
| triethanolamine | 1104 | 89 | 849 | 55 | 1.30 | 0.02 | 0.14 | 1.28 |
| phosphate | 1185570 | 83693 | 903078 | 95472 | 1.31 | 0.02 | 0.14 | 1.28 |
| 2-deoxytetronic acid | 70465 | 6230 | 91667 | 6482 | 0.77 | 0.02 | 0.14 | 1.27 |
| 2-ketoisocaproic acid | 58219 | 7178 | 43219 | 6164 | 1.35 | 0.02 | 0.14 | 1.26 |
| 3-4-hydroxyphenylpropionic acid | 13758 | 1948 | 8218 | 950 | 1.67 | 0.02 | 0.14 | 1.25 |
| kynurenic acid | 7616 | 862 | 5105 | 548 | 1.49 | 0.02 | 0.14 | 1.25 |
| beta-alanine | 367673 | 49181 | 520786 | 51430 | 0.71 | 0.02 | 0.14 | 1.25 |
| **Urine PND 51** |  |  |  |  |  |  |  |  |
| glyceric acid | 145497 | 20718 | 72226 | 6126 | 2.01 | < 0.01 | 0.08 | 3.15 |
| galactonic acid | 596148 | 70135 | 307452 | 39949 | 1.94 | < 0.01 | 0.18 | 2.85 |
| pinitol | 21446 | 3184 | 9947 | 2494 | 2.16 | < 0.01 | 0.18 | 2.74 |
| sorbitol | 1736910 | 393770 | 501582 | 127076 | 3.46 | < 0.01 | 0.18 | 2.73 |
| shikimic acid | 41127 | 3579 | 24807 | 5064 | 1.66 | < 0.01 | 0.23 | 2.59 |
| conduritol-beta-expoxide | 18051 | 5840 | 39408 | 7063 | 0.46 | < 0.01 | 0.23 | 2.57 |
| alanine-alanine | 26981 | 4237 | 51057 | 9975 | 0.53 | 0.01 | 0.32 | 2.44 |
| pantothenic acid | 107600 | 6942 | 72473 | 8408 | 1.48 | 0.01 | 0.32 | 2.38 |
| melibiose | 2134 | 321 | 1064 | 134 | 2.01 | 0.01 | 0.32 | 2.37 |
| ribonic acid | 29486 | 3692 | 18103 | 2631 | 1.63 | 0.02 | 0.44 | 2.25 |
| parabanic acid | 70019 | 6665 | 47615 | 5257 | 1.47 | 0.02 | 0.44 | 2.23 |
| 3-hydroxyphenylacetic acid | 5739 | 1041 | 3126 | 297 | 1.84 | 0.03 | 0.63 | 2.06 |
| tartaric acid | 7696 | 2633 | 12239 | 2074 | 0.63 | 0.03 | 0.63 | 2.06 |
| linoleic acid | 8510 | 2273 | 3284 | 720 | 2.59 | 0.03 | 0.66 | 2.02 |
| xanthurenic acid | 1029 | 141 | 694 | 93 | 1.48 | 0.04 | 0.67 | 1.99 |
| galactitol | 436656 | 98408 | 224033 | 42085 | 1.95 | 0.04 | 0.68 | 1.93 |
| raffinose | 7932 | 960 | 5381 | 1197 | 1.47 | 0.05 | 0.68 | 1.89 |
| mucic acid | 1808 | 161 | 1159 | 120 | 1.56 | 0.05 | 0.68 | 1.87 |
| 2,6-diaminopimelic acid | 1533 | 128 | 2755 | 565 | 0.56 | 0.05 | 0.68 | 1.87 |
| 4-pyridoxic acid | 17770 | 1435 | 12949 | 1476 | 1.37 | 0.05 | 0.68 | 1.87 |
| 3-hydroxybenzoic acid | 2733 | 792 | 14304 | 6869 | 0.19 | 0.05 | 0.68 | 1.86 |
| alanine | 271248 | 37269 | 494645 | 143531 | 0.55 | 0.05 | 0.70 | 1.83 |

^1^Mean of normalized (mTIC) peak intensities (mz/rt) for human milk (HM) or milk formula (MF) after MetaboAnalyst analyses. Sample size were n = 25 per group at PND 21 and n = 15 per group at PND 51.

^2^SEM = Standard error of the mean

^3^Fold change of HM mean

^4^*P*-Value ≤ 0.05

^5^FDR = Benjamini-Hochberg false discovery rate adjusted P-Value

^6^VIP = variable importance in projection, in PLS-DA models using all annotated metabolites to compare HM and MF. The table only presents metabolites with significant differences between diet groups; all detected metabolites are provided in Supplemental dataset S1.
